# Floral and inflorescence ontogeny in the *Brownea *clade (Fabaceae: Detarioideae):insights into a morphologically diverse lineage of legumes

**DOI:** 10.1007/s10265-026-01723-2

**Published:** 2026-07-20

**Authors:** Ana Tereza Novaes Parga Rodrigues, Marcus José de Azevedo Falcão, Vidal de Freitas Mansano

**Affiliations:** 1 Instituto de Pesquisas Jardim Botânico do Rio de Janeiro, DIPEQ, Rua Pacheco Leão 915, Jardim Botânico, Rio de Janeiro, RJ 22460–030 Brazil; 2 Universidade do Estado do Rio de Janeiro, IBRAG-DBV, Rua São Francisco Xavier, 524, Maracanã, Rio de Janeiro, RJ 20550-900 Brazil

**Keywords:** Floral development, *Heterostemon*, Leguminosae, *Macrolobium*, *Paloue*

## Abstract

The subfamily Detarioideae stands out among legumes as a clade with diverse floral morphology. Within it, the seven genera of the Brownea clade exhibit significant variability, especially in the merism of corolla and androecium, inflorescence architecture, and pollination strategies. This study aims to expand the understanding of the morphology and development of inflorescences and flowers of six species within the clade: *Brownea longipedicellata*, *Brownea leucantha*, *Heterostemon mimosoides*, *Macrolobium suaveolens*, *Paloue paraensis* and *Paloue speciosa*. We also provided an anatomical analysis of the nectariferous hypanthium of four of these species*.* We discuss the roles of heterochrony and mechanical pressures in floral development and how these factors lead to the observed morphological diversity. The study reveals several ontogenetic novelties for the Brownea clade, such as the basipetal development of *B. longipedicellata* racemes, the erratic organogenesis of the flowers of *B. leucantha* due to mechanical pressure from adjacent flowers in the congested inflorescences, the heteromorphic development of the corolla in *H. mimosoides,* and the development of massive bracteoles of *M. suaveolens* impacting the development of subsequent whorls. We provide new insights on the meristic reduction in different taxa within Detarioideae. In addition, we observed that in the species of the clade *Heterostemon*+*Macrolobium*+*Paloue*, the adaxial petal surrounds the carpel in the bud, a previously undescribed feature in Detarioideae, and possibly a new synapomorphy of this clade. We describe the inflorescence development in the group, which varies from simple racemes in most taxa to large capituliform racemes in *Browneopsis* and in some species of *Brownea* and *Paloue*. We compare this inflorescence and floral diversity, including the anatomy of the hypanthium with different pollination syndromes, including entomophily, ornithophily, and chiropterophily.

**Supplementary Information:**

The online version contains supplementary material available at https://doi.org/10.1007/s10265-026-01723-2.

## Introduction

Fabaceae is the third-largest family of angiosperms, comprising six subfamilies (Lewis et al. 2005; LPWG 2017). Among these, the pantropical Detarioideae stands out as the third largest subfamily, with 76 genera and 815 species (Estrella et al. 2018; LPWG 2024). The Neotropics account for around 30% of this diversity, many of which are of notable ecological, economic, medicinal and cultural importance (Estrella et al. 2018; Klitgaard 1991; Lewis et al. 2005).

Members of Detarioideae exhibit remarkable diversity in floral morphology and symmetry (Bruneau et al. 2014; Citeli et al. in prep; Cowan and Polhill 1981a, 1981b; Kochanovski et al. 2018; Krüger et al. 1999; Pedersoli et al. 2010; Prenner and Cardoso 2017; Tucker 2000a, 2000b, 2001a, 2002a, 2003c). This diversity includes deviations from the typical legume floral groundplan with five sepals, five petals, two whorls of five stamens each, and a single carpel (LPWG 2017; Tucker 1987). In most Detarioideae, the calyx is pentamerous in early-stage development, but the two adaxial sepals fuse in later stages, giving the appearance of a tetramerous calyx at anthesis (Bruneau et al. 2014; Ferguson and Tucker 1994). The majority of taxa in Detarioideae (70% of genera and 64% of species) exhibit reductions in the number of petals and/or stamens (present work), with those being lost or reduced to petal rudiments or staminodes (Tucker 1988, 1989a, 2000d). Rarely, even sepals are absent (Tucker 2000a).

Besides this diversity, some floral characters are widespread among most Detarioideae and are even possible synapomorphies for the subfamily or, at least, for most of its clades as the aforementioned fusion of adaxial sepals, the style which curves toward the adaxial side of the flower in bud, and a staminal ring at the base of the filaments (Bruneau et al. 2014; Estrella et al. 2018; Prenner and Cardoso 2017). Concerning bracteoles, its morphology and function has historically played a significant role in classifying the groups now included within Detarioideae such as former tribes Cynometreae, Amherstieae l.s., and Macrolobieae (Breteler 1995; Cowan and Polhill 1981a; Léonard 1957). However, molecular phylogenetic studies by Bruneau et al. (2000) recovered them as paraphyletic*.* Tucker (2000a, 2002b) proposed two morphologies of the floral meristem during bracteole initiation to distinguish taxonomic groupings: the circular type, associated with smaller bracteoles; and the omega type, which have bracteoles well-developed shortly after initiation. However, subsequent phylogenies revealed this character as homoplastic across different lineages (e.g. Bruneau et al. 2014). Bracteoles can play different roles, such as attracting pollinators, protecting the flower bud (Breteler 1995; Cowan and Polhill 1981a, 1981b), and mechanically influencing the flower development (Tucker 2000a, 2002a, 2002b).

**Fig. 1:**
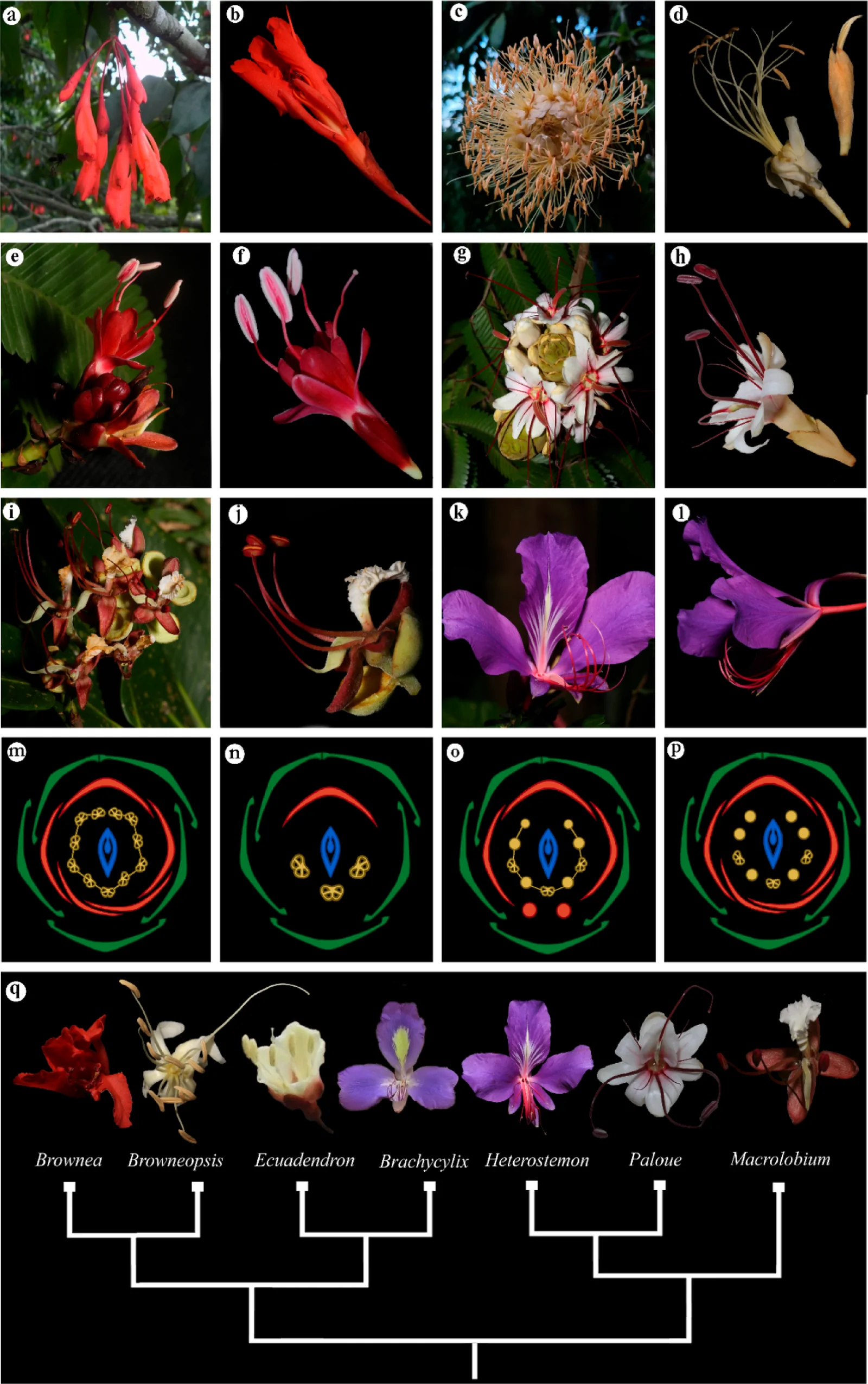
Flowers and inflorescences of the studied genera: **a–b** Inflorescence and flower of *B. longipedicellata.***c–d** Inflorescence and flower of *B. leucantha*, with the details of its bracteoles. **e–f** Inflorescence and flower of *P. speciosa*. **g–h** Inflorescence and flower of *P. paraensis*. **i–j** Inflorescence and flower of *Macrolobium latifolium* Vogel (a morphologically similar species to the studied *M. suaveolens*, included here for illustrative purposes). **k–l** Flowers of *H. mimosoides*. **m** Floral diagram of *B. longipedicellata* and *B. leucantha*. **n** Floral diagram of *M. suaveolens*. **o** Floral diagram of *H. mimosoides*. **p** Floral diagram of *P. paraensis* and *P. speciosa*. **q** Cladogram of the Brownea clade and the phylogenetic relationships of its genera based on the topology obtained by Schley et al 2018. Credits: Photo of *Brachycylix*: Emilio Constantino; Photo of *Ecuadendron*: Michaël Moens; Photos of the other species: Marcus Falcão

Detarioideae is currently classified into six tribes (Estrella et al. 2018). Among these, Amherstieae is the largest, but intra- and intergeneric relationships within the group remain often poorly supported (Baker et al. 2022; Estrella et al. 2018; Zuntini et al. 2024). The morphologically diverse flowers of Amherstieae are characterized by the large, persistent bracteoles in most genera (Estrella et al. 2018). Meristic reduction and morphological modifications leading to zygomorphy are common, as is the differentiation of the adaxial petal, often more prominent than the others (Cowan and Polhill 1981a, 1981b). Within Amherstieae, the Brownea clade is also morphologically diverse, having its current circumscription comprising seven genera: *Brownea* Jacq., *Browneopsis* Huber, *Brachycylix* (Harms) R. S. Cowan, *Ecuadendron* D.A. Neill, *Heterostemon* Desf., *Macrolobium* Schreb., and *Paloue* Aubl. (Fig. 1), all of which are Neotropical (Bruneau et al. 2000; Redden et al. 2006, 2010). Despite its diversity, with about 130 spp. Floral ontogeny studies have been conducted for only two species within the clade: *Brownea latifolia* Jacq. and *Macrolobium acaciifolium* (Benth.) Benth. (Tucker 2000b, 2002b). However, Tucker’s study on *Macrolobium* lacks images, and much of the commentaries focus on *Macrolobium crassifolium* A. Chev., which was later recombined as *Anthonotha crassifolia* (Baill.) J. Léonard. (Tucker 2002b), an unrelated African species.

A key feature of most taxa in the Brownea clade is the reduction in the number of petals and stamens (Cowan 1953, 1975, 1976; Klitgaard 1991; Neill 1998; Redden and Herendeen 2006; Schley et al. 2018). Also, the fusion of bracteoles into a tube is considered a synapomorphy for the clade (Redden et al. 2010). However, most species of *Macrolobium* display free bracteoles (Cowan 1953; Murphy et al. 2018). Table 1 provides a comparative analysis of the main floral morphological characteristics across the clade's genera.

**Table 1:** Comparison of the main variations in floral morphology among the genera of the Brownea clade

Taxon	Symmetry	Bracteoles	Corolla	Androecium	Inflorescence
*Brachycylix*^1^	Strongly zygomorphic	Small, fused	3 developed, 2 petal rudiments	5 stamens; 4 staminodes; forms a sheath	Pendulous, long, lax
*Brownea*^2^*	Radial (usually)	Large, fused	4–5 petals, almost equal, erect	10–12; forms a sheath	Erect to pendulous; usually globose, congested
*Browneopsis*^2^	Radial (usually)	Absent	3–5 petals, subequal, erect or reflexed	9–26; forms a sheath	Erect; globose, congested
*Ecuadendron*^3^	Slightly zygomorphic	Medium, fused	5 petals, the adaxial larger	5 stamens; 4 staminodes; fused at the base	Pendulous, long, lax
*Heterostemon*^4^*	Strongly zygomorphic	Small, fused	3 developed, 2 petal rudiments	3 stamens; 6 staminodes; forms a sheath	Erect, short, lax
*Macrolobium*^5^*	Strongly zygomorphic	Large, free to partially fused	1 petal, rarely 2–4 petal rudiments	3, free, rarely staminodes	Usually erect, rarely pendulous; short, lax
*Paloue*⁶*	Slightly to strongly zygomorphic	Large, fused	1, 3, or 5 developed petals, unequal or equal; 0, 2, or 4 petal rudiments	9 stamens or 3 stamens and 6 staminodes; fused at the base	Erect; long to short, lax to congested; rarely globose racemes

Sources: (^1^) = Cowan 1975, (^2^) = Klitgaard 1991, (^3^) = Neill 1998, (^4^) = Cowan 1976, (^5^) = Cowan 1953, (⁶) = Redden et al. 2018; Bracteole size is quoted in comparison to flower size and not in absolute measurements; (*): Genera studied in the present work

Most of the 22 species of *Brownea* have red, radially symmetrical and commonly pseudotubular flowers with fused bracteoles, five petals, and a staminal sheath. Their inflorescences are commonly capituliform racemes, sometimes cauliflorous (Klitgaard 1991). The species *Brownea longipedicellata* Huber, *Brownea angustiflora* Little, *Brownea birschellii* Hook.f. and *Brownea coccinea* Jacq. stand out with their loose racemes, which generally bear fewer flowers with long pedicels. *Browneopsis*, a sister genus with eight species, is similar to *Brownea* in its inflorescence arrangement. However, its flowers exhibit white to slightly greenish or yellowish hues. The androecium ranges from nine to more than twenty stamens (Klitgaard 1991). *Paloue* comprises 16 species, varying in floral symmetry, corolla and androecium reduction, and inflorescence types. Flower colors range from red or pink to white, with two predominant morphologies: 1) 1 or 3 developed petals and a greater number of stamens; 2) 5 developed petals and an androecium commonly reduced to three stamens and six staminodes. The species can also vary from lax inflorescences with a few flowers to capituliform racemes with dozens of flowers. *Heterostemon*, a closely related genus, comprises seven species and has large and pink, purple, or white flowers, featuring three petals, two rudimentary petals, three stamens and six staminodes with antherodes arranged in a monadelphous sheath. *Macrolobium*, also closely related, is the most rich genus of the Brownea clade, including 78 species (Murphy et al. 2018; POWO 2024). Its flowers exhibit relatively conserved morphology, with the corolla reduced to a single adaxial petal and the androecium with three stamens (Cowan 1953). The flowers of *Brachycylix* resemble those of *Heterostemon* due to some shared corolla and androecium characteristics (Table 1). However, *Brachycylix* also shares similarities with *Ecuadendron*, particularly in having five developed stamens and by the long and pendulous racemes (Cowan 1975; Neill 1998).

Given the above, we see that the floral diversity of those genera focus mainly on the corolla and androecium whorls. The calyx and the gynoecium are much more stable morphologically (Cowan 1976; Klitgaard 1991; Neill 1998) although there are exceptions, like *Macrolobium* which can, for example, have five free sepals in some species (Cowan 1953; Prenner and Cardoso 2017; Tucker 2000a, 2002b).

The hypanthium in flowers of taxa in the Brownea clade vary in width, length, and depth. All species are likely nectar producers, a trait common in the subfamily (Cowan and Polhill 1981a). Investigating the nectariferous nature of their hypanthia could yield valuable insights into the pollination mechanisms of these taxa. However, no anatomical studies have yet explored their microstructural differences or their relationship to pollination. Different pollination syndromes have been documented for taxa within clade (Arroyo 1981; Banks and Klitgaard 2000; Banks and Rudall 2016; Fleming et al. 2009; Knudsen and Klitgaard 1998). According to Cowan and Polhill (1981a), the floral diversity in Detarioideae reflects an evolutionary history of coadaptation with animals for defense and reproduction.

Thus, this study aims to perform ontogenetic and anatomical analyses of flowers and inflorescences of a morphologically diverse set of six species from five genera of the Brownea clade. We seek to address key evolutionary questions while contributing to the systematics and understanding of floral evolution of this fascinating group of Fabaceae.

## Materials and methods

**Fig. 2:**
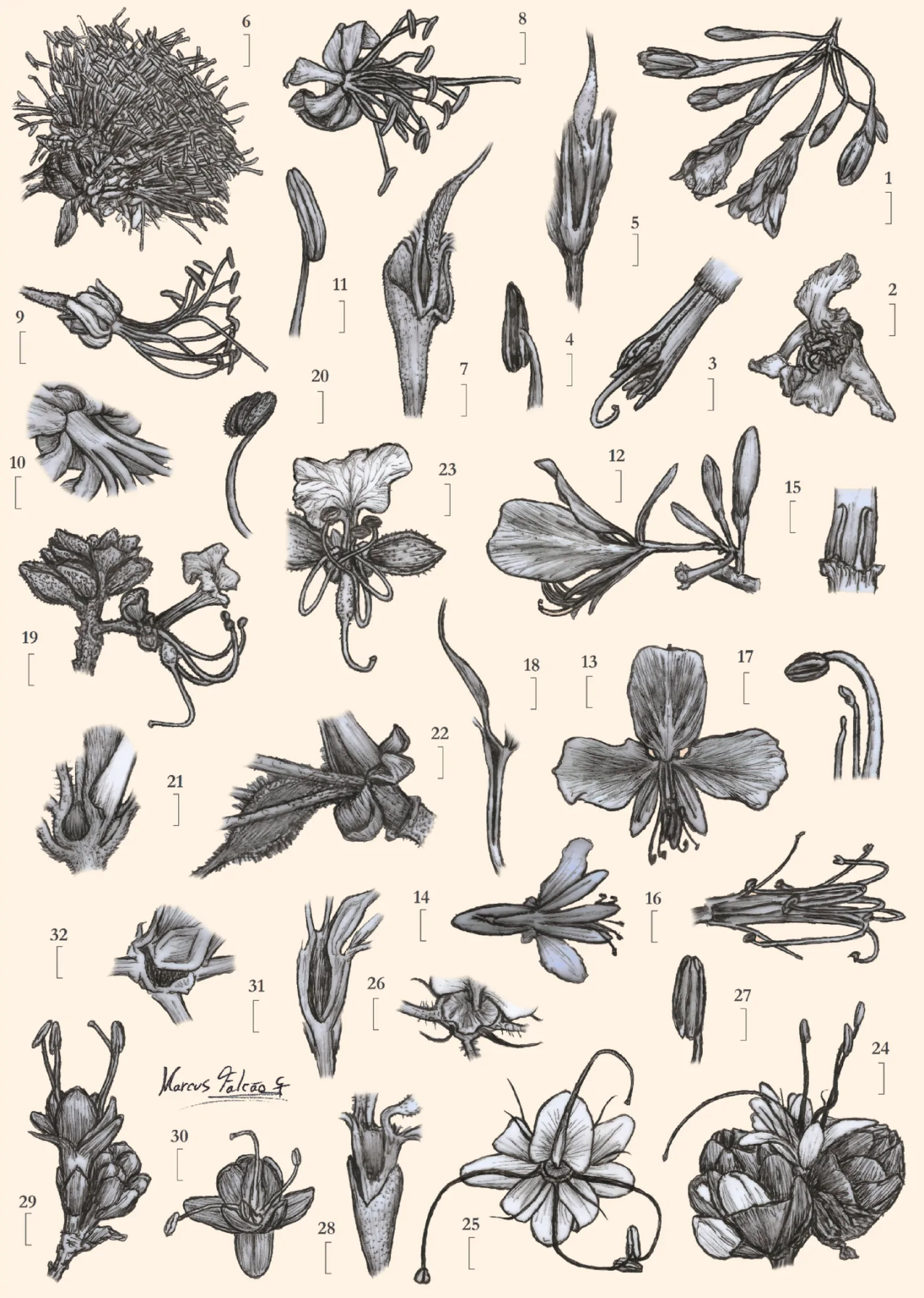
Illustration of inflorescences and flowers of the studied species. **1–5***B. longipedicellata*. **6–11***B. leucantha*. **12–18***H. mimosoides*. **19–23***M. suaveolens*. **24–28***P. paraensis*. **29–32***P. speciosa*. **1, 6, 12, 19, 24, 29** Inflorescence. **2, 8, 13, 23, 25, 30** Polar view of the flower. **3, 10, 16** Detail of staminal sheath. **4, 11, 20, 27** Detail of the anther. **5, 7, 18, 21, 28, 31** Detail of the longitudinally sectioned hypanthium with stipe adnate to its adaxial wall. **9** Lateral view of the flower. **14** Abaxial view of the flower. **15** Detail of the two reduced petals. **17** Detail of anther, medium antheroid (middle one) and small antheroid (left one). **22** Lateral detailed view of the flower with reduced sepals. **26, 32** Detailed polar view of the hypanthium aperture and staminal ring showing staminodes. Scale bars: **1, 12** = 1 cm; **2, 3, 16, 28, 30** = 7 mm; **4, 20** = 0.6 mm; **5, 10, 11, 26, 27, 31** = 4 mm; **6** = 2 cm; **7, 18, 29** = 8 mm; **8, 14, 24** = 1.2 cm; **9, 13 25** = 1.5 cm; **15, 32** = 3 mm; **17, 19, 23** = 2 mm; **21, 22** = 1 mm; **30** = 6 mm. Drawn by Marcus Falcão

Six species from four genera were selected for analysis in this study (Figs. 1, 2): *B. longipedicellata* (Fig. 1a, b) (*Novaes AT 02*), *Brownea leucantha* Jacq. (Fig. 1c, d) (*Falcão MJ 96*), *Heterostemon mimosoides* Desf. (Fig. 1k, l) (*Novaes AT 01*), *Macrolobium suaveolens* Spruce ex Benth. (Fig. 1i, j) (*Pinto RB 467*), *Paloue paraensis* (Ducke) Redden (Fig. 1g, h) (*Falcão MJ 362*) and *Paloue speciosa* (Ducke) Redden (Fig. 1e, f) (*Novaes AT 03*). These two species of *Paloue*, although having relatively similar flower morphologies, were selected due to their different pollination syndromes and inflorescence morphology. The first five species were collected at the Arboretum of the Rio de Janeiro Botanical Garden, while the latter was collected during an expedition to the state of Pará in 2014. Voucher specimens were deposited in the RB and HSTM herbaria.

For ontogenetic analysis, the collected samples were fixed in FAA 70 (5% formaldehyde, 5% glacial acetic acid, and 90% of 70% ethanol; Johansen 1940) and stored in 70% ethanol. The floral materials were dissected and observed under a stereomicroscope (Leica MZ7.5). After dissection, the samples were dehydrated in an alcohol series (Tucker 1993), subjected to critical point drying (Leica EM CPD 030), and then fixed onto metal stubs with double-sided carbon tape. The samples were coated with a gold-palladium alloy using a sputter coater (Emitech K550X) and observed under a scanning electron microscope (JEOL JSM-6490LV-LABNANO-CBPF) (Tucker 2000b, 2000c).

Anatomical analyses of the flowers were conducted on floral buds fixed in 2.5% glutaraldehyde and 0.1M sodium phosphate buffer at pH 7.2. These buds were then dehydrated in an alcohol series, embedded in glycol-methacrylate resin (Gabriel 1982; Gerrits et al. 1991), sectioned transversely and longitudinally using a rotary microtome (Leica RM 2245), stained with 0.05% Toluidine Blue, and observed and photographed under a light microscope (Olympus BX50) (O’Brien et al. 1964). Anatomical studies of the hypantial region were conducted for four species: *B. longipedicellata*, *H. mimosoides*, *P. paraensis* and *P. speciosa*.

Terminology for organography and organogenesis follows Tucker (1987, 2000a, 2000c, 2002a, b, 2003b) and Harris and Harris (1994). For anatomical structure, the terminology follows Appezzatto-da-Gloria and Carmello-Guerreiro (2006) and Tolke et al. (2020).

## Results

The organographies, including descriptions of the development of inflorescences and flowers, along with corresponding figures, are organized in alphabetical order of the species: *B. longipedicellata* (Figs. 3, 4), *B. leucantha* (Figs. 5, 6), *H. mimosoides* (Figs. 7, 8), *M. suaveolens* (Figs. 9, 10), *P. paraensis* (Figs. 11, 12) and *P. speciosa* (Figs. 13, 14). The description of the anatomical structure of the nectaries follows as the final section (Fig. 15).

**Fig. 3:**
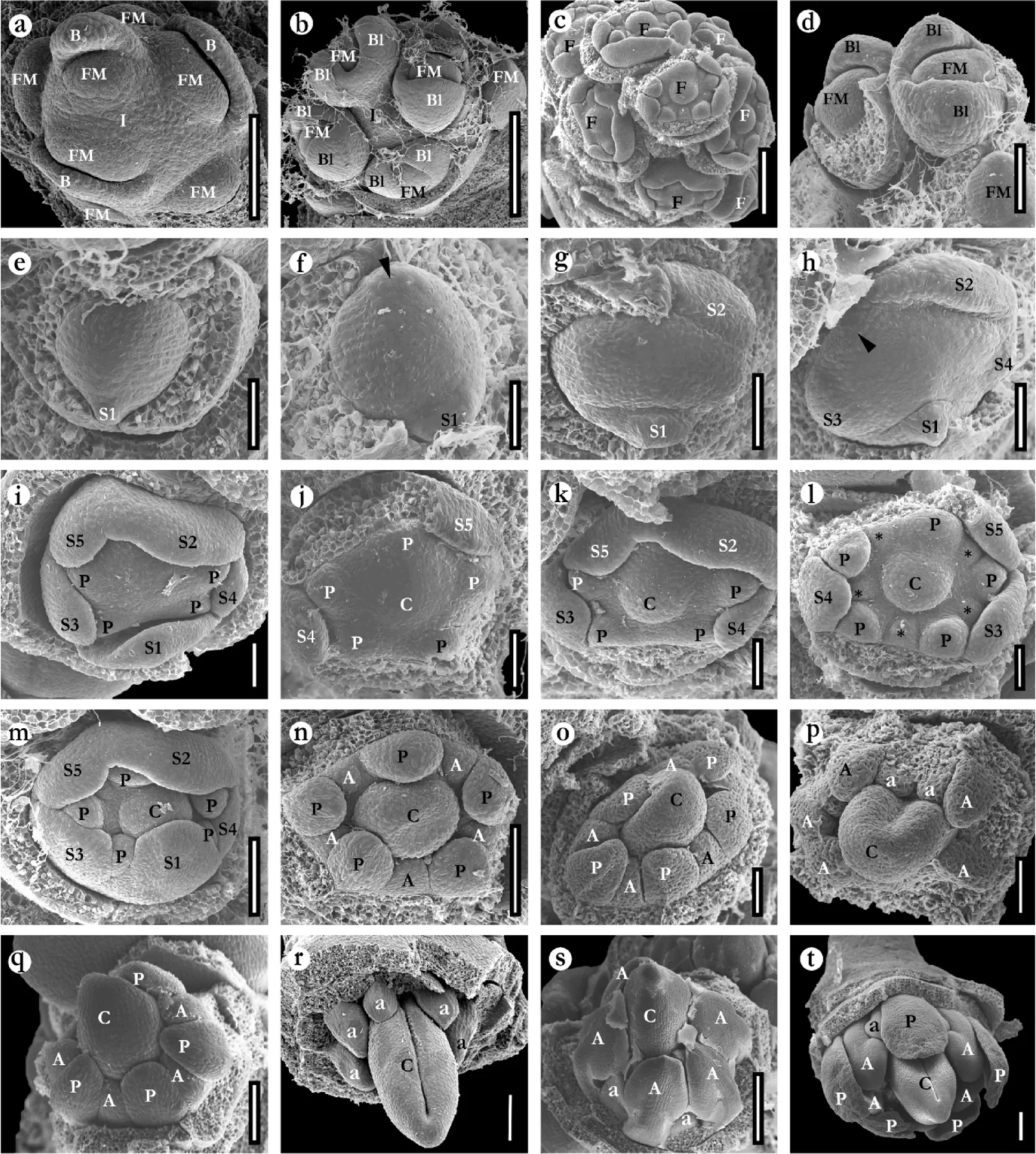
Development of flowers and inflorescences of *B. longipedicellata* (SEM). All type 1 bracts and some type 2 bracts were removed in **a**. All bracts were removed in **b** and **d**. All bracts and bracteoles were removed in **c** and **e–t**. **a** Inflorescence (I) with bracts (B) subtending undifferentiated floral meristems (FM). **b** Floral meristems (FM) at very similar stages and surrounded by bracteoles (Bl). **c** End of organogenesis, with apical flowers (F) more developed than basal ones. **d** Lateral view of two meristems (FM) paired by bracteoles (Bl). **e** Initiation of the first median abaxial sepal (S1). **f** Protuberance formed by the pressure of the bracteoles on the adaxial region (arrow). **g** S1 and S2. **h** The first four sepals (S1, S2, S3 e S4) and the possible initiation of S5 (arrow). **i** S2 and S5 fused and petals (P) evident (except adaxial). **j** Carpel (C) initiation (S1, S2 and S3 removed). **k** Petals (P) and carpel (C) more elongated (S1 removed).** l** Petals (P) with different development rates and primordia of antesepalous (asterisk) stamens (S1 and S2 removed). **m** Sepals covering the bud. **n** Carpel (C) begins to show in its adaxial portion the beginning of the formation of the carpel cleft and antesepalous stamens (A) visible. **o** Petals (P) and antesepalous stamens (A) covering the innermost part. **p** Carpel (C), antesepalous (A) and antepetalous stamens (a), drawing attention to the pair of antepetalous stamens opposite the adaxial petal that was removed and to the carpel cleft in formation. **q** Petals (P) differentiating and carpel (C) apex tapering. **r** Carpel (C) cleft almost closed and antesepalous stamens (A) beginning to differentiate. **s–t** Antesepalous stamens (A) differentiated into anther and filament. **t** Petal (P) base narrowing. Scale bars: **a, e–l, o, p** = 50 μm; **b–d, m, n, q, r, t** = 100 μm; **c, s** = 200 μm

**Fig. 4:**
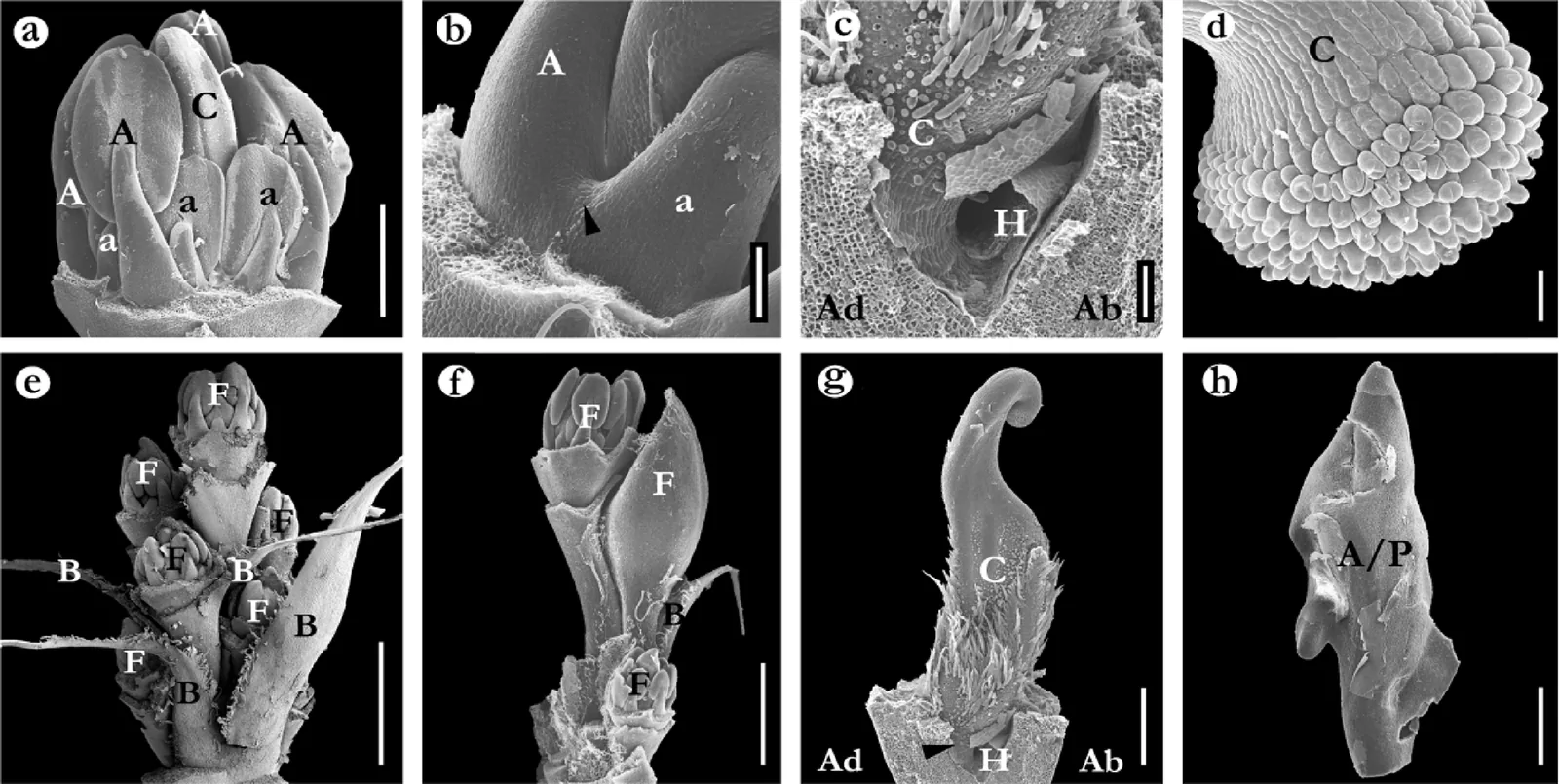
Late stages of development in inflorescences and flowers of *B. longipedicellata* (SEM). **a** Adaxial side of the bud with sepals and petals removed to show differentiated stamens (A, a) and carpels (C) and “extra” stamen. **b** Detail of the base of two fused filaments (arrow) between antesepalous (A) and antepetalous (a) stamens. **c** Detail of the developing hypanthium (H) and stipe of the carpel (C) adnate to the adaxial (Ad) wall, opposed to the abaxial (Ab) side. **d** Detail of the carpel (C) stigma differentiating. **e** Inflorescence with oblong and filiform bracts (B) and flower (F) buds in late stages. **f** Inflorescence showing the elongated pedicel of the buds and an entire flower (F) bud to show the fit that forms between them. **g** Carpel (C), showing its differentiation into the ovary, stipe adnate to the adaxial (Ad) wall of the hypanthium (H) (arrow), style curved to the abaxial side (Ab), and stigma. **h** Petaloid stamen (A/P) removed from a flower in preanthesis. Scale bars: **d** = 50 μm; **b, c** = 100 μm; **a, g, h** = 500 μm; **e, f** = 1 mm

**Fig. 5:**
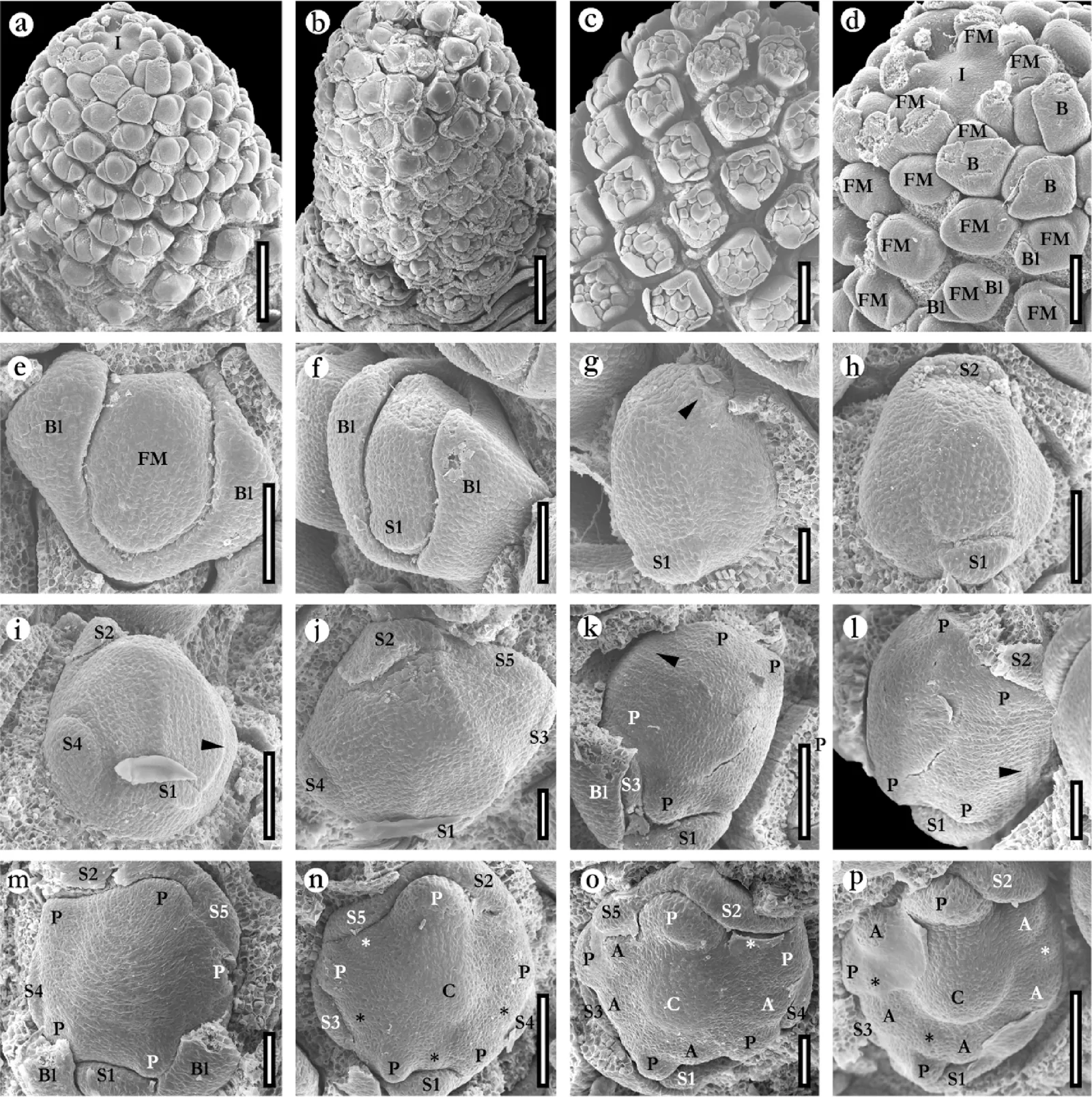
Development of flowers and inflorescences of *B. leucantha* (SEM). Bracts were completely removed in **b–c** and **e–p** and partially removed in **a** and **d**. Bracteoles were completely removed in **b–c** and **g–p** and partially removed in **a** and **d**. **a–c** Lateral view of inflorescences, from youngest to oldest. **d** Detail of the apex of the same inflorescence (I) as in **a**, with detail of the floral meristems (FM), bracts (B) and bracteoles (Bl). **e** Floral meristem (FM) with elongating bracteoles (Bl). **f** Initiation of S1. **g** Protuberance in the adaxial position formed by the pressure of bracts and bracteoles (arrow). **h** Initiation of S2. **i** Polar view slightly displaced to the abaxial side, showing S1, S2, S4 and possibly S3 (arrow). **j** Dorsoventrally flattened meristem, with S1, S2, S3, S4 and S5 initiating. **k** Slightly lateralized polar view showing the 5 initiated petals (P), but with different developments, and S5 not being visible (arrow). **l** Same bud as in **k**, but from another angle, showing that S4 is not visible (arrow) (S2 partially cut). **m** Bud with all sepals (S) and petals (P) present. **n** Initiated carpel (C) and signs of antesepalous stamens (asterisk). **o** Antesepalous stamens visible (A) and one antepetalous stamen emerging (asterisk). **p** Antepetalous abaxial stamens emerging (asterisk). Scale bars: **g, j, l, m, o** = 50 μm; **e, f, h, i, k, n, p** = 100 μm; **d** = 200 μm; **a–c** = 500 μm

**Fig. 6:**
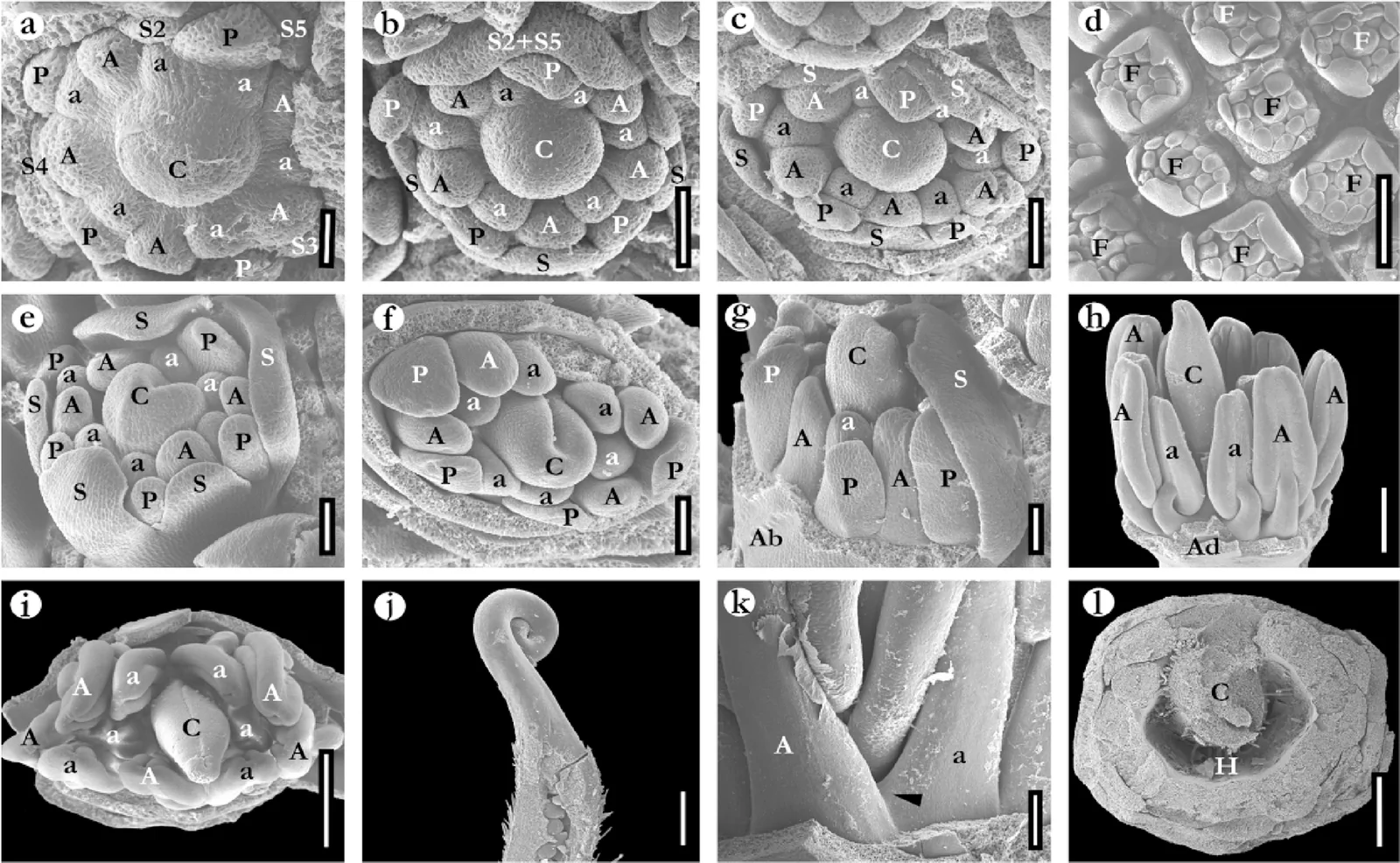
Late stages of development in flowers of *B. leucantha* (SEM). Bracts and bracteoles removed in **a–l**. Polar view of floral buds in **a–f**, **i** and **l**, with the abaxial side down. Lateral view of buds and floral structures in **g, h**, **j, k**. **a–c** All sepals (S), petals (P), carpel (C), antesepalous stamens (A) and antepetalous stamens (a) initiated and elongating with petal differentiating at **b** and beginning of carpelar cleft formation at **c**. **d** Floral (F) buds at very similar stages of elongation. **e** Fusion of the sepals (S) bases and elongating organs. **f** Dorsoventrally flattened bud with 10 stamens (A, a) instead of 11. **g** Organs differentiating. **h** Sepals (S) and petals (P) removed, antesepalous (A) and antepetalous (a) stamens well differentiated and carpel (C) with tapered and slightly curved apex. **i** Bud flattened dorsoventrally, petals (P) and sepals (S) removed, stamens differentiated and of different sizes. **j** Carpel (C) differentiated with an open ovary showing ovules, style elongated and curving towards the abaxial side and differentiated stigma. **k** Detail of the base of the filaments of an antesepalous (A) and an antepetalous (a) stamen merging (arrow). **l** All organs removed to show the hypanthium (H) forming and the stipe of the carpel (C) adnate to the adaxial wall. Scale bars: **a** = 50 μm; **b, c, e–g** = 100 μm; **k** = 200 μm; **d, i, j, l** = 500 μm

**Fig. 7:**
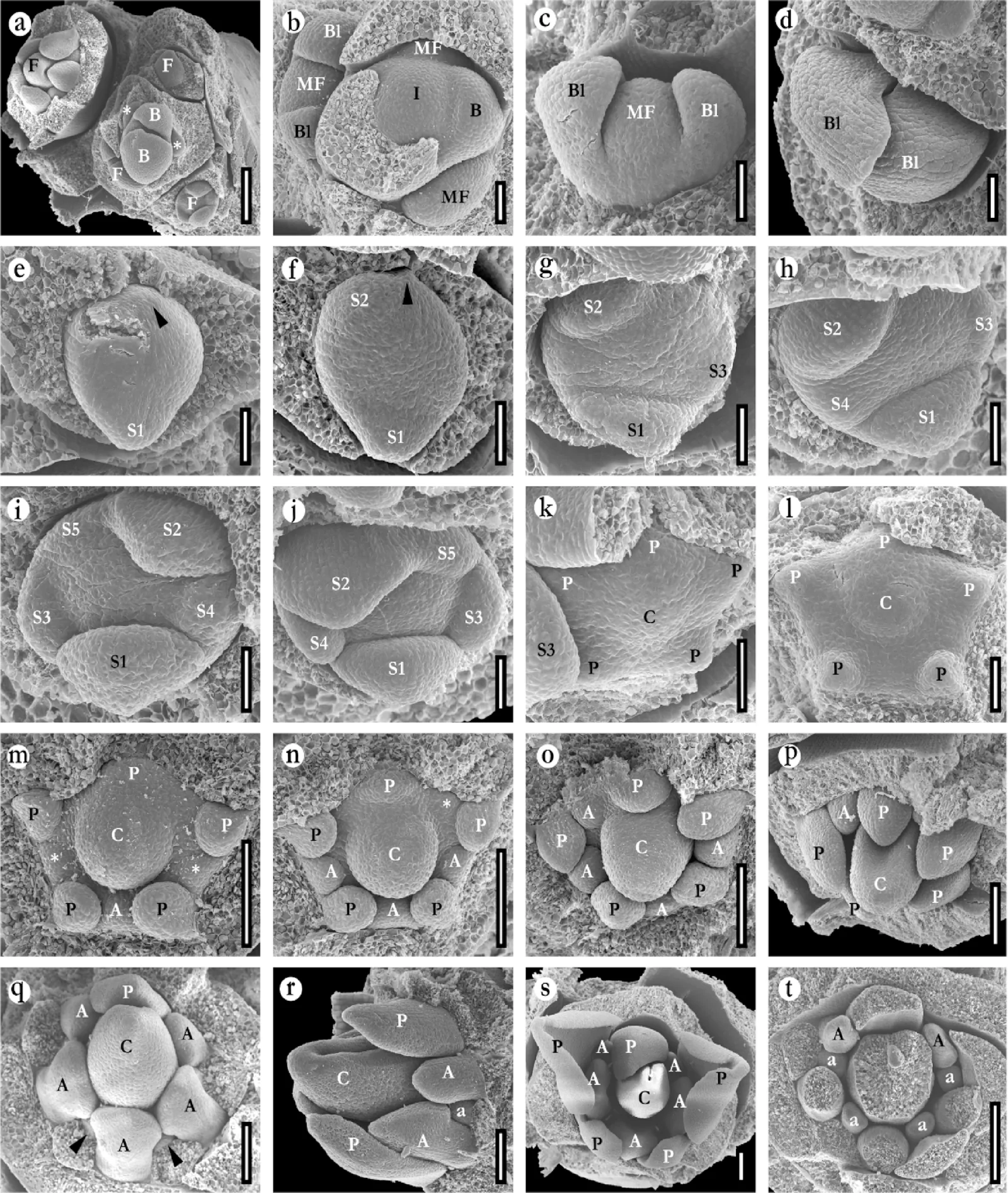
Development of inflorescence and flowers of *H. mimosoides* (SEM). Bracts were removed totally or partially in **a–t**. Bracteoles were removed in **e–t**. Sepals were removed in **k–t**. Petals were removed totally or partially in **q, r** and **t**. Polar view of flower buds in **d–t**. **a** Inflorescence showing different stages of meristem development (asterisk) and flower (F) buds with bracts (B) or opened. **b** Meristematic apex of the inflorescence (I), with detail to floral meristems with bracteoles (Bl). **c** Lateral view of a meristem (FM) with two elongating bracteoles (Bl). **d** Bracteoles (Bl) covering the meristem. **e** S1 emerging and highlighting the bulge in the median adaxial position (arrow). **f** S2 emerging and highlighting the bulge in the median adaxial position (arrow). **g** S3 emerging. **h** S4 emerging. **i** All sepals (S) initiated with S5 emerging. **j** All sepals (S) initiated, highlighting S2 and S5 fused. **k** Petals (P) in evidence and the carpel (C) emerging in the center. **l** Petals (P) with different development rates. **m** Abaxial antesepalous stamen (A) and laterals antesepalous stamen emerging (asterisk), petals (P) elongating. **n** Abaxial and lateral antesepalous stamens (A) visible and adaxial ones emerging (asterisk). **o** Petals (P), carpel (C) and antesepalous stamens (A) elongating. **p** Petals (P) differentiating and beginning of carpel (C) cleft formation. **q** Antesepalous stamens (A) differentiating and antepetalous already emerged (arrow). **r** Lateral antepetalous stamen appearing (a), adaxial antesepalous stamens (A) with different sizes. **s** Lateral petals (P) larger, adaxial petal (P) surrounding the carpel (C). **t** Lateral and abaxial antesepalous stamens, petals and carpel removed to show antepetalous stamens (a) and adaxial antesepalous stamens (A). Scale bars: **b–l** = 50 μm; **m–s** = 100 μm; **a, t** = 200 μm

**Fig. 8:**
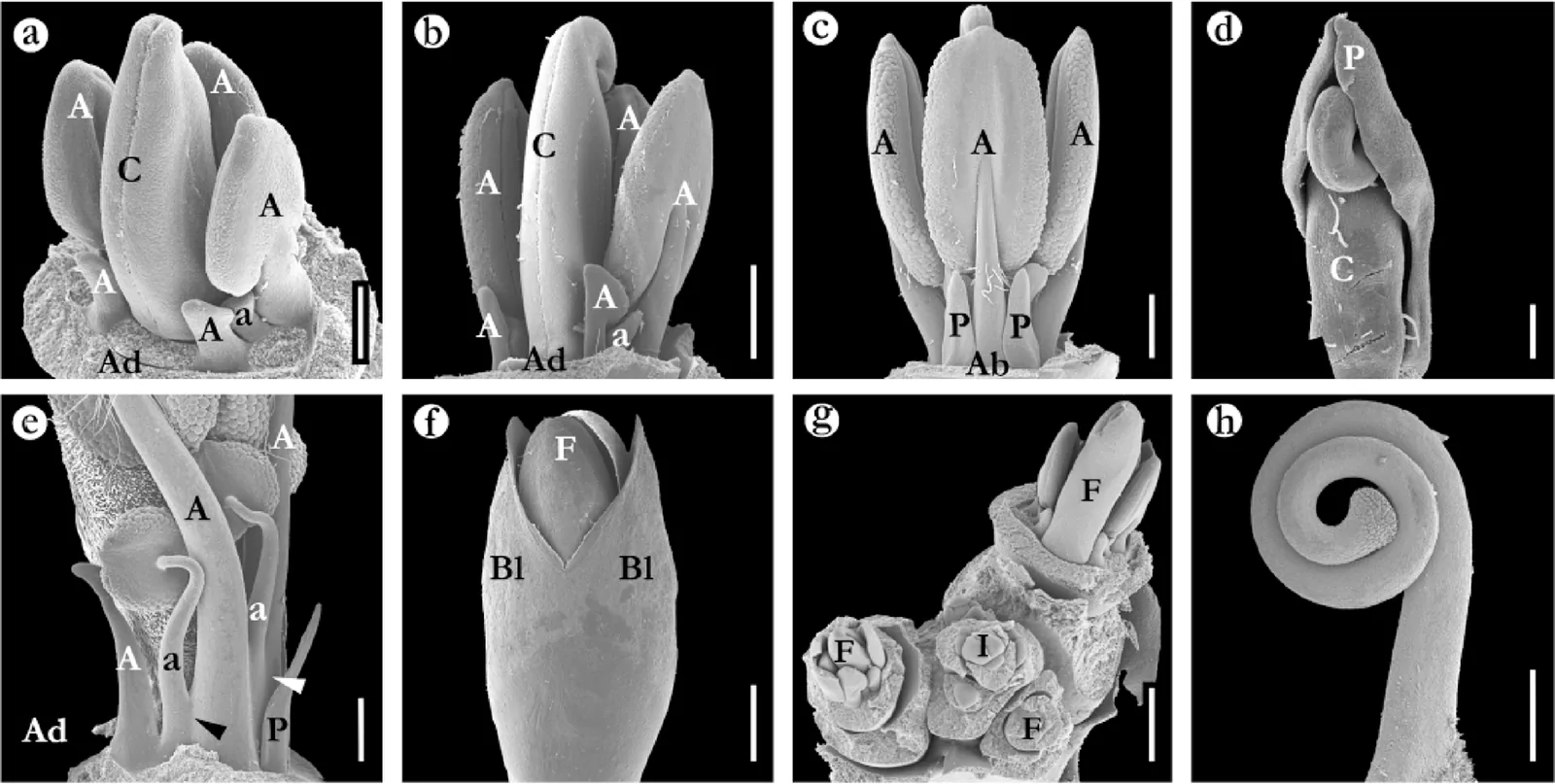
Development of inflorescence and flowers and details of floral structures of *H. mimosoides* (SEM). Bracts removed in **a–h**. Bracteoles and sepals removed in **a–e** and **g, h**; Petals completely or partially removed in **a–e** and **g, h**. Lateral view of buds or floral structures in **a–f** and **h**. **a–b** Adaxial side (Ad) of two different stages, highlighting the lateral and abaxial antesepalous stamens (A) that are much more developed than the adaxial ones and the antepetalous stamen (a), in addition to the apex of the carpel (C) curved toward the abaxial side. **c** Abaxial side (Ab) showing the abaxial reduced petals (P) compared to the antesepalous stamens (A). **d** Abaxial side of the detail of the carpel (C) being enveloped by the adaxial petal (P). **e** Formation of the monadelphous sheath (arrow) with heteromorphic antesepalous (A) and antepetalous (a) stamens and a reduced petal (P). **f** Entire flower (F) bud with partially fused bracteoles (Bl). **g** Polar view of the inflorescence (I) showing different stages of floral (F) development. **h** Detail of the style forming a spiral and differentiated stigma. Scale bars: **a, d** = 200 μm; **b, c**, **e–h** = 500 μm

**Fig. 9:**
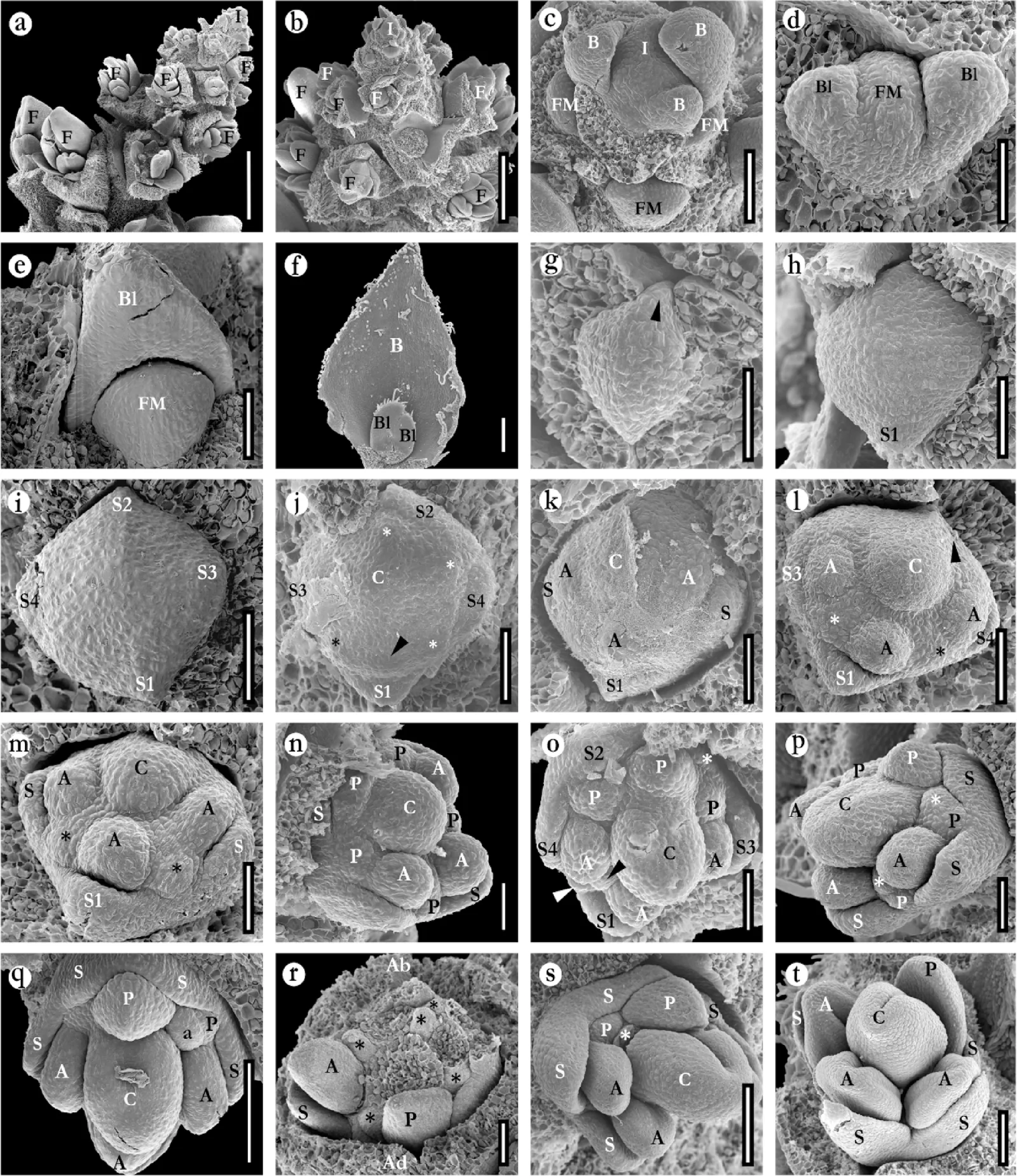
Development of inflorescences and flowers of *M. suaveolens* (SEM). Bracts were removed in whole or in part in **a–e** and **g–t**. Bracteoles were removed in whole or in part in **a, b** and **g–t**. Lateral view of inflorescence and flower bud in **a**, **e, f** and **n–q**. Polar view of inflorescence and flower buds in **b, c**, **h–m** and **r–t**. Abaxial side facing downward in **d** and **g–t**. **a, b** Inflorescences (I) at different angles with several flower (F) buds at different stages of development. **c** Apex of inflorescence (I) with detail to floral meristems (FM) and developing bracts (B). **d** Floral meristem (FM) with two developing bracteoles (Bl). **e** One bracteole was removed to show the size ratio of the bracteole (Bl) to the meristem (FM). **f** Bract (B) with bud with closed bracteoles (Bl). **g** Protuberance formed by the pressure of the bracteoles (arrow). **h** S1 emerging. **i** S2, S3 and S4 emerging. **j** Petals (asterisks), carpel (C) and abaxial antesepalous stamen (arrow) emerging. **k** Sepals (S) and abaxial and lateral antesepalous stamens (A) around the carpel (C). **l, m** Abaxial and lateral antesepalous stamens (A) more developed than petals (asterisks) and S2 is barely visible (arrow). **n** Petals (P) visible and highlight on the unevenness of the bud. **o** Highlight on S5 (asterisk), adaxial and lateral petals (P) visible, abaxial petal (white arrow) and one abaxial antepetalous stamen (black arrow). **p** Sepals (S), antesepalous stamens (A) and carpel (C) elongating, adaxial petal (P) developing more than the others and antepetalous stamen primordia (asterisk). **q** Adaxial side, only one side with petal (P) and antepetalous stamen (a) primordia, in addition to carpel (C) cleft forming. **r** Adaxial (Ad) side facing towards and abaxial (Ab) upwards with organs removed, remaining a sepal (S), one antesepalous stamen (A) and adaxial petal (P) to show undeveloped primordia of dubious nature (asterisk).** s** Formation of carpel (C) cleft and primordia of one lateral petal (P) and antepetalous stamen (asterisk). **t** Antesepalous stamens (A), sepals (S) and adaxial petal (P) differentiated, and carpel (C) apex slightly curved. Scale bars: **d–f, h–p**, **r** = 50 μm; **c, q, s, t** = 100 μm; **g** = 200 μm; **b** = 500 μm; **a** = 1 mm

**Fig. 10:**
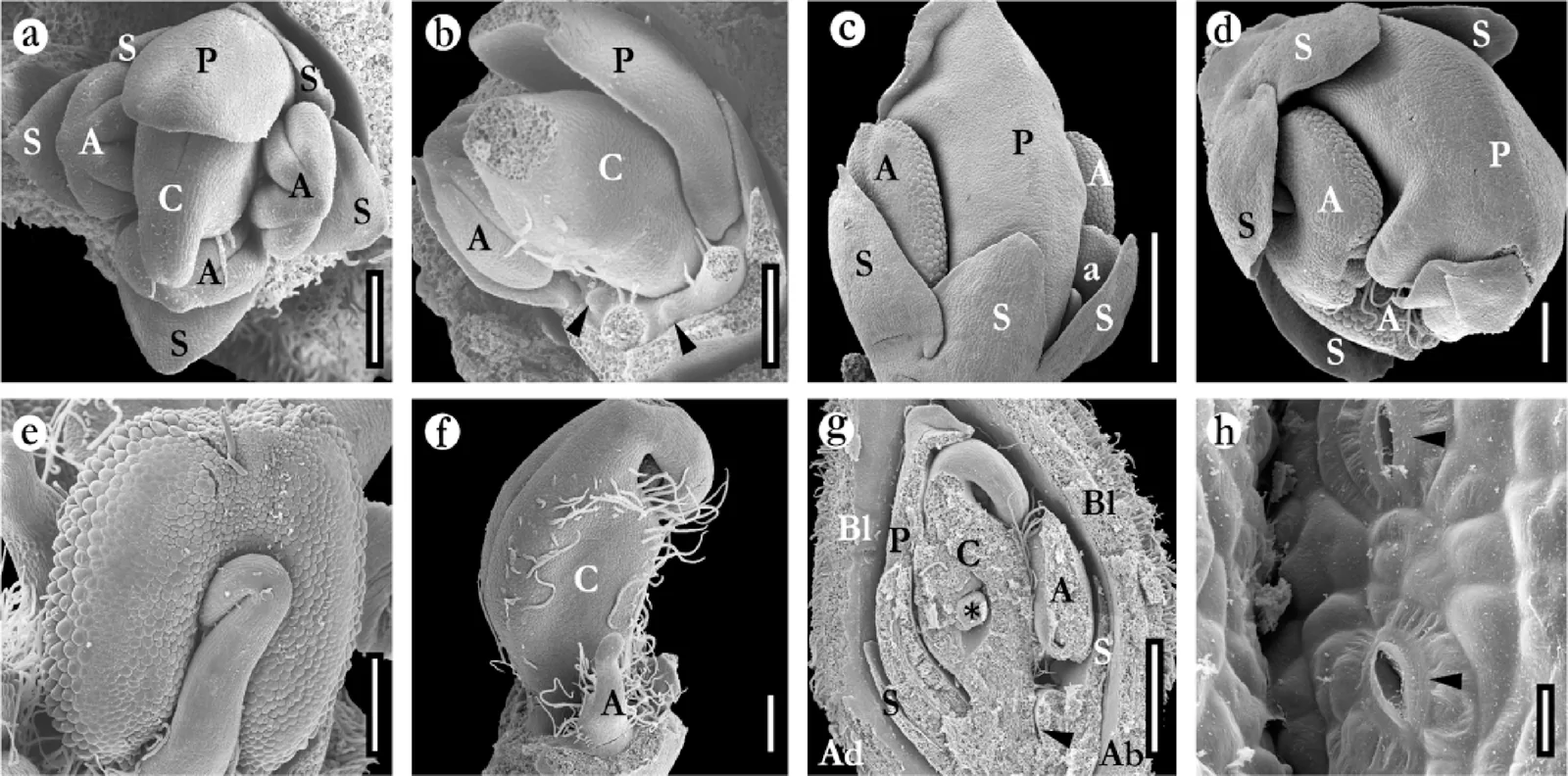
Floral development and details of structures of *M. suaveolens* (SEM). Bracts completely removed and bracteoles completely or partially removed in **a–g**, Sepals completely or partially removed in **b** and **e–g**. Polar view of flower buds in **a, b** and **d**, with abaxial side facing downwards. Lateral view of flower buds in **c** and **e–h**. **a** Stamens (A), adaxial petal (P) and carpel (C) larger than sepals (S) and formation of the style. **b** Organs removed, remaining a stamen (A), adaxial petal (P) carpel (C) and undeveloped primordia (arrow). **c** Adaxial petal (P) covering the carpel (C) and a fourth stamen (A) that developed on the right side. **d** Adaxial petal (P) embracing the carpel from above. **e** Detail of the surface of a differentiated anther. **f** Detail of the carpel (C) with marginal trichomes, style curved toward the abaxial side, other flower structures removed, except for the filament of a stamen (A). **g** Adaxial side (Ad) on the left and abaxial side (Ab) on the right side of the image, longitudinal section of a bud with bracteoles (Bl), adaxial petal (P), antesepalous stamen (A) and carpel (C), showing the formation of the hypanthium (arrow) and an ovule in the ovary (asterisk). **h** Detail of stomata (arrow) on the inner surface of the bracteole. Scale bars: **h** = 10 μm; **a, b, d–f** = 200 μm; **c, g** = 500 μm

**Fig. 11:**
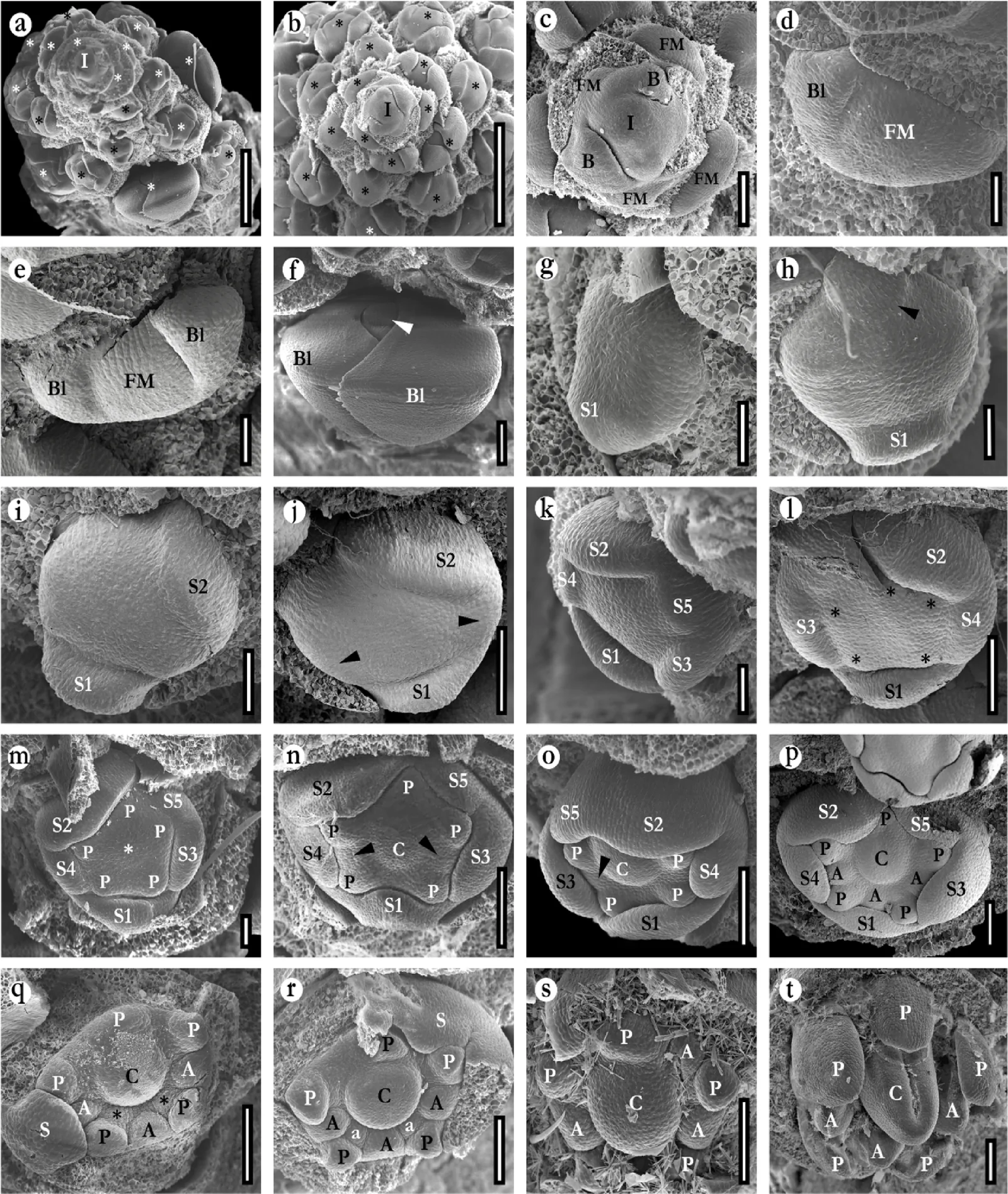
Development of inflorescences and flowers of *P. paraensis* (SEM). Bracts were removed totally or partially in **a–t**. Bracteoles were removed totally or partially in **a, b** and **g–t**. Sepals were removed totally or partially in **q–t**. Polar view of inflorescences in Fig. **a–c** and of meristems/flower buds in **d–t**. **a, b** Different inflorescences (I) showing the proximity of numerous flower buds (asterisk) to each other. **c** Detail of the inflorescence (I) apex with bracts (B) and floral meristems (FM) differentiating. **d** Floral meristem (FM) with one bracteole (Bl) formed and another forming (arrow). **e** Floral meristem (FM) with two bracteoles (Bl) formed. **f** Bracteoles (Bl) covering the meristem, except for the gap in the median adaxial region (arrow). **g** S1 emerging. h Protuberance formed by the pressure of the bracteoles (arrow). **i** S2 initiated. **j** Initiation of S3 and S4 simultaneously (arrow). **k** All sepals (S) initiated and S5 fusing to S2. **l** Petals initiating (asterisk). **m** Petals (P) initiated and carpel initiating (asterisk). **n** Initiation of lateral antesepalous stamens (arrow). **o** Sepals (S), petals (P), and carpel (C) elongating and initiation of lateral antesepalous stamen. **p** Bud with sepals (S), petals (P), carpel (C) and initiated abaxial and lateral antesepalous stamens (A). **q** Initiation of antepetalous abaxial stamens (asterisk), poorly developed adaxial petal (P). **r** Sepals (S), petals (P), carpel (C), abaxial and lateral antesepalous stamens (A) and abaxial antepetalous stamens (a). **s** Bud with artifacts that hinder visualization, but petals (P), antesepalous stamens (A) and carpel (C) can be seen. **t** Petals (P) and antesepalous stamens elongating and carpel (C) cleft forming. Scale bars: **d, e, g–i, k** = 50 μm; **c, f, j, l–t** = 100 μm; **a, b** = 500 μm

**Fig. 12:**
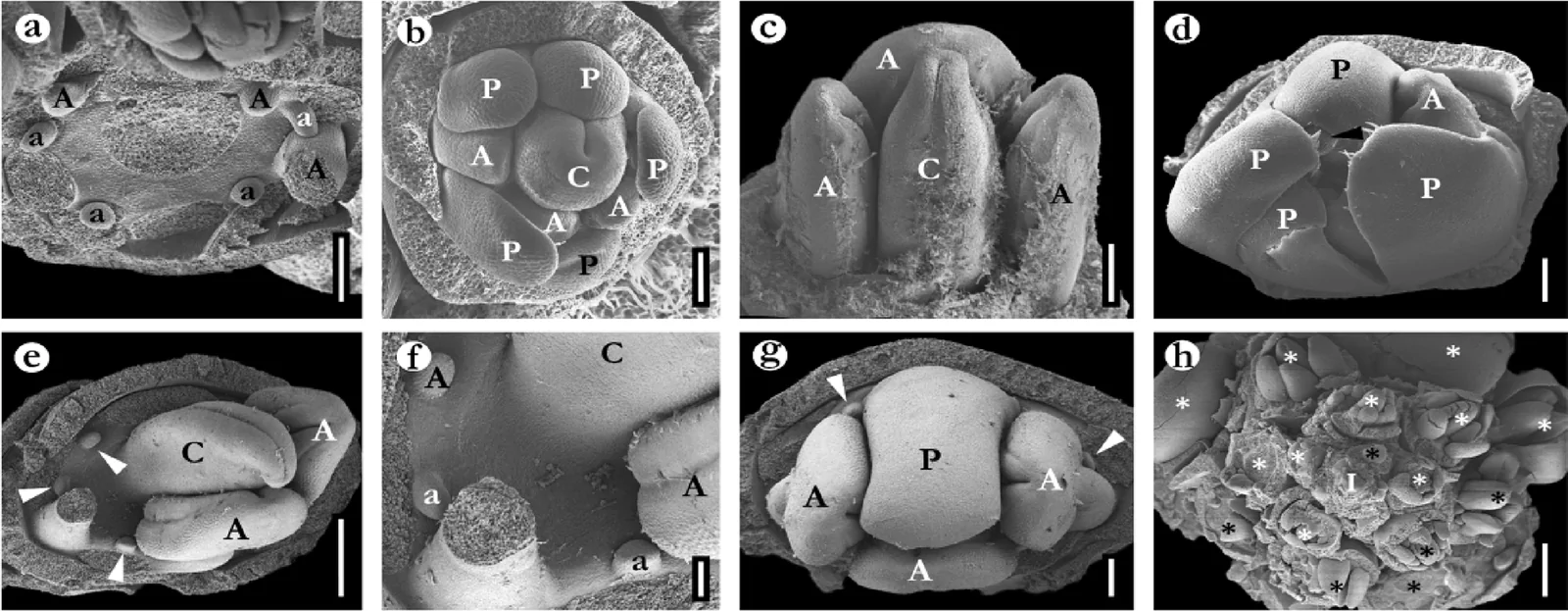
Development of flowers and inflorescences and details of floral structures of *P. paraensis* (SEM). Bracts, bracteoles, and sepals completely or partially removed in **a–h**. Petals completely or partially removed in **a, c, e–g**. Polar view of flower buds in **a, b**, **d–g**, with the abaxial side facing downwards, and of the inflorescence in **h**. **a** Organs removed to show poorly developed adaxial antesepalous stamens (A) and four antepetalous stamens (a). **b** Differentiated petals (P) and adaxial petal “touching” the carpel (C). **c** Adaxial lateral view of a bud with differentiated antesepalous stamens (A) and style of carpel (C) curving towards the abaxial side. **d** Four imbricated petals (P), one of the lateral ones has been removed where can be seen an antesepalous stamen (A), and the innermost petal is the adaxial one, enveloping the carpel (C). **e** Lateral stamen removed to show traces of poorly developed stamens (arrow) and the hypanthium forming. **f** Detail of adaxial antesepalous stamen (A) and antepetalous stamens (a) poorly developed. **g** Vestigial stamens (arrow) and adaxial petal (P) surrounding the carpel (C). **h** Inflorescence (I) with numerous stages of floral development and spiral arrangement of buds (asterisk). Scale bars: **b, f** = 100 μm; **a, c, d, g** = 200 μm; **e, h** = 500 μm

**Fig. 13:**
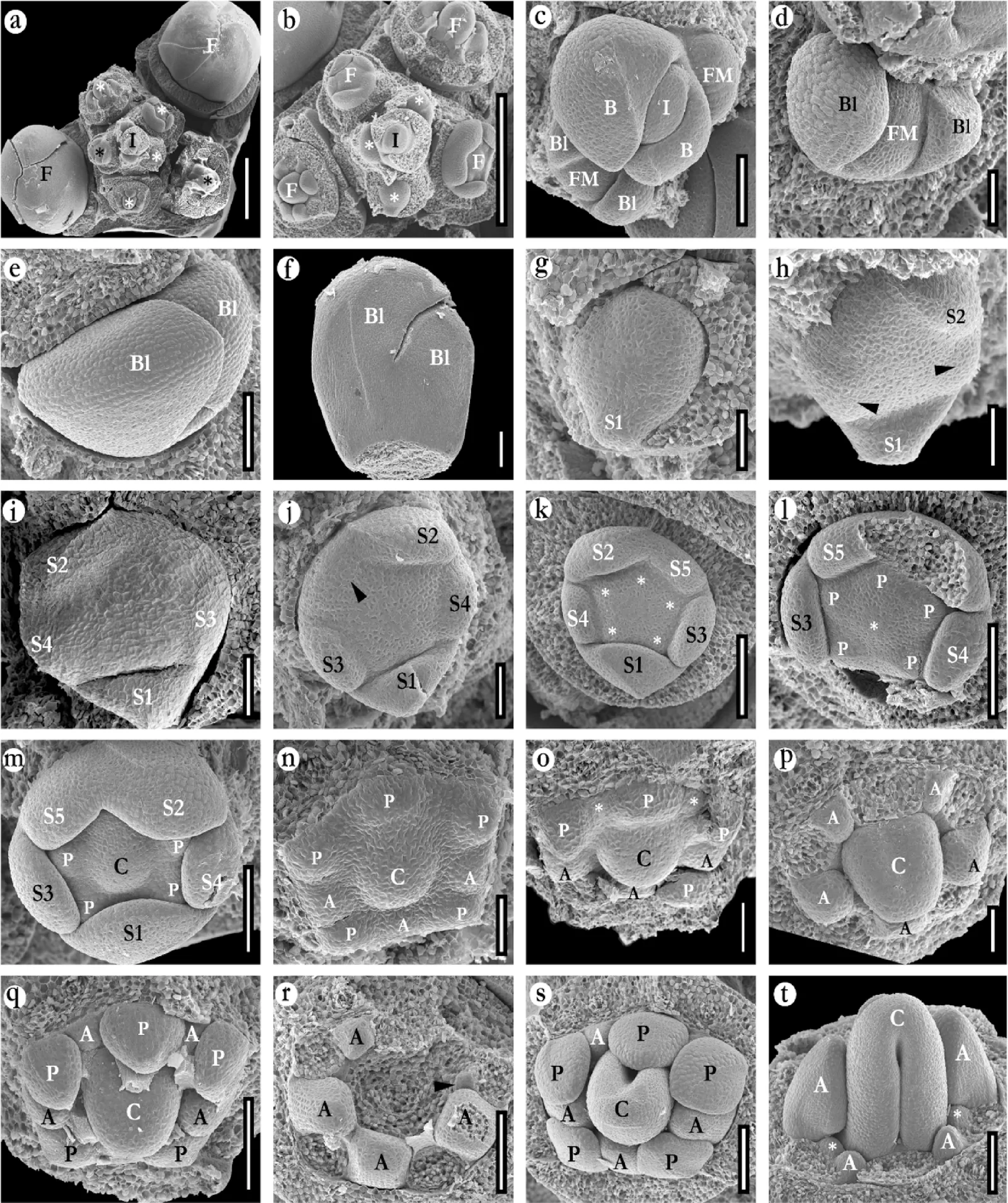
Development of inflorescences and flowers of *P. speciosa* (SEM). Bracts were removed in whole or in part. Bracteoles were removed in whole or in part in **a, b** and **g–t**. Sepals were removed in whole or in part in **l** and **n–t**. Polar view of inflorescences in **a–c** and of meristems/flower buds in **d, e** and **g–s**, with abaxial side facing downwards. **a** Inflorescence (I) with different stages of floral development (F, asterisk). **b** Detail of inflorescence of **a**. **c** Inflorescence (I) apex with elongating bracts (B) and floral meristems (FM) with bracteoles (Bl). **d** Floral meristem (FM) with a pair of bracteoles (Bl). **e** Bracteoles (Bl) covering the meristem and leaving a gap in the abaxial position (arrow). **f** Closed flower bud with overlapping bracteoles (Bl). **g** Initiation of S1. **h** S2 initiated, and S3 and S4 initiating (arrows). **i** Initiation of S3 and S4. **j** S5 initiating (arrow). **k** All sepals initiated (S1, S2, S3, S4 and S5) and petals initiating (asterisk) and S5 fused to S2. **l** Sepals (S) and petals (P) Carpel initiating (asterisk). **m** Sepals (S), petals (P), and carpel elongating (C). **n** initiation of abaxial and lateral antesepalous stamens (A). **o** Petals (P), carpel (C), antesepalous stamens (A) and initiation of adaxial antesepalous stamens (asterisk). **p** Antesepalous stamens (A) and carpel (C) forming carpel cleft. **q** Petals (P) differentiating around antesepalous stamens (A) and carpel (C). **r** Antesepalous stamens (A) and one lateral antepetalous stamen visible (arrow). **s** Petals (P), antesepalous stamens (A) and carpel (C) differentiating. **t** Adaxial lateral view of bud without petals showing lateral antesepalous stamens (A) differentiating while the two adaxial ones (A) are poorly developed, and two antepetalous stamens (asterisk). Scale bars: **d, g–j, n–p** = 50 μm; **c, e, f, k–m, q–t** = 100 μm; **a, b** = 500 μm

**Fig. 14:**
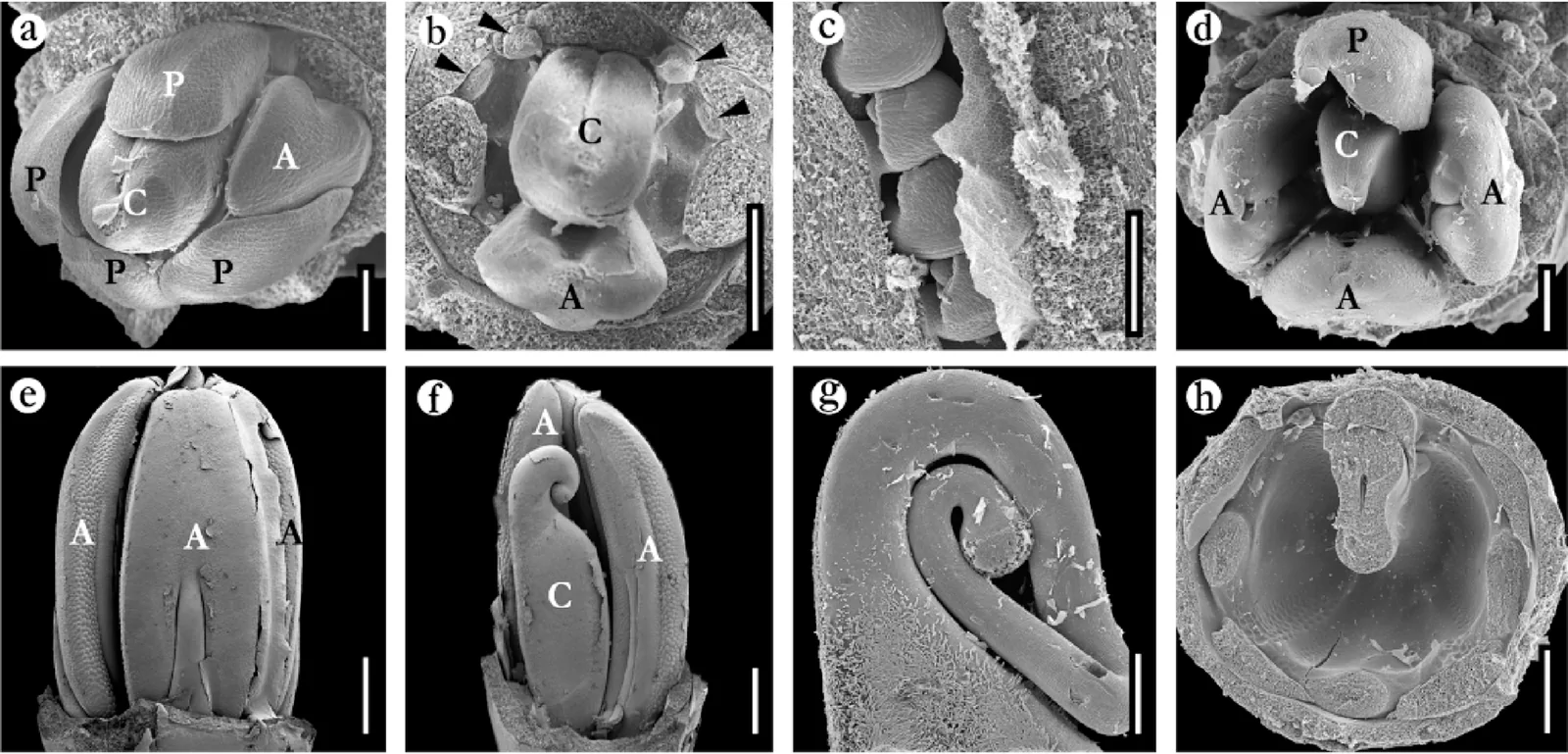
Final stages of floral development and detail of structures of *P. speciosa* (SEM). Bracts, bracteoles, and sepals removed. Petals completely or partially removed in **b–h**. Polar view of flower buds in **a, b, d** and **h**, with the abaxial side facing downwards. Lateral view of flower buds and structures in **c**, **e–g**. **a** Petals (P), carpel (C) and one antesepalous stamen differentiating into anther and filament. **b** One abaxial antesepalous stamen, carpel (C) and staminodes (arrow). **c** Detail of ovules. **d** Antesepalous stamens (A), adaxial petal (P) surrounding the carpel (C) and style curving towards the abaxial side. **e** Abaxial side of bud showing developed antesepalous stamens (A). **f** Style of the carpel (C) curving. **g** Detail of style forming spiral and differentiated stigma. **h** Whorls removed to show hypanthium and stipe adnate to the adaxial wall. Scale bars: **a** = 100 μm; **b–d** = 200 μm; **e–h** = 500 μm

**Fig. 15:**
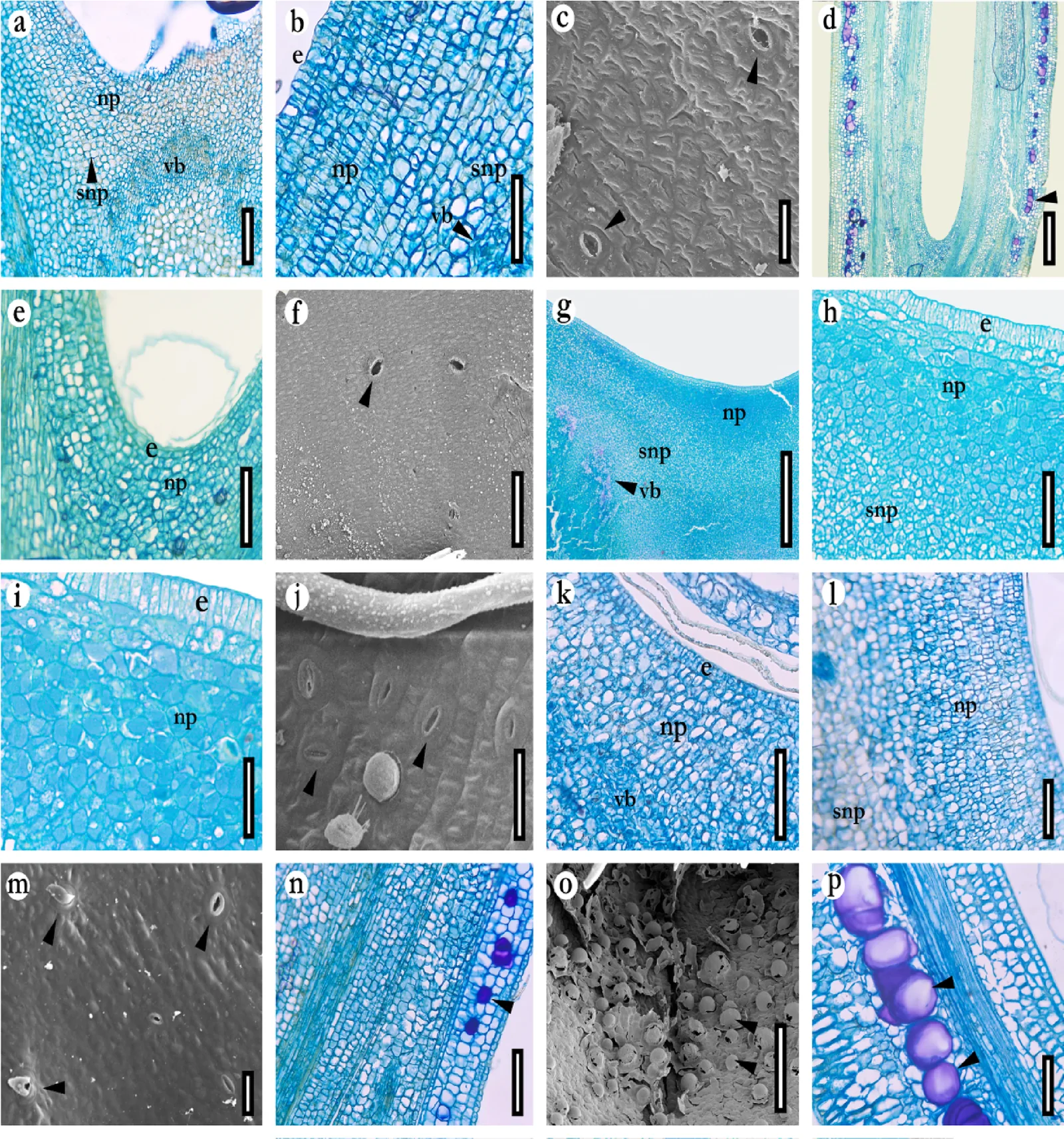
Anatomical structure of the nectariferous tissue of the hypanthium of *B. longipedicellata, H. mimosoides, P. paraensis* and *P. speciosa*. **a, b**, **d, e, g–i, k, l, n, p** are images taken from light microscopy of longitudinal sections of flower buds at pre-anthesis. In the case of *B. longipedicellata*, *P. paraensis*, and *P. speciosa*, the sections are perpendicular to the dorsoventral axis of the bud and to *H. mimosoides*, the sections are parallel to the dorsoventral axis of the bud. Fig. **c, f, j, m**, **o** are scanning electron micrographs. *B. longipedicellata* in **a–c**. **a** General view of the hypantial region with nectariferous parenchyma (np), subnectariferous parenchyma (snp) and vascular bundle (vb). **b** Detail of the nectariferous parenchyma (np) and epidermis (e). **c** Stomata on the surface of the hypanthium (arrow). *H. mimosoides* in **d–f**. **d** General view of the hypantial region with a highlight to the presence of idioblasts (arrow). **e** Detail of the nectariferous parenchyma (np) and epidermis (e). **f** Stomata on the surface of the hypanthium (arrow). *P. paraensis* in **g–j**. **g** General view of the hypanthium region with nectariferous parenchyma (np), subnectariferous parenchyma (snp) and vascular bundles (vb). **h** Color difference between the nectariferous parenchyma (np) and the subnectariferous parenchyma (snp). **i** Detail of the nectariferous parenchyma (np) and epidermis (e). **j** Stomata on the surface of the hypanthium (arrow). *P. speciosa* in **k–m**. **k** Nectariferous parenchyma (np), vascular bundle (vb) and epidermis (e). **l** Color difference between the nectariferous parenchyma (np) and the subnectariferous parenchyma (snp). **m** Stomata on the surface of the hypanthium, some of them with unidentified substances at the operculum (arrow). **n** Detail of the idioblasts of *B. longipedicellata* (arrow). **o** Detail of the modified trichomes of *B. longipedicellata* (arrows). **p** Detail of the idioblasts of *P. speciosa* (arrows). Scale bars: **c**, **i, j**, **m** = 20 μm; **a, b**, **e**, **h**, **k, l**, **n**, **p** = 50 μm; **d**,** f**,** o** = 100 μm;** g** = 500μm

### ***B. longipedicellata*** (Figs. 1a, b, m, 2.1–5, 3a–t, 4a–h, 15a–c, n, o)

#### Inflorescence

Organography—Simple, pendulous, terminal, axillary, or cauliflorous raceme. The peduncle is approximately 1 cm long, typically bearing seven to ten flowers, though occasionally up to 15. There are two types of bracts: Type (1) corresponds to the proximal bracts; these are ovate, overlapping, pubescent, alternate, and distichous, and in most cases do not subtend any flowers except for the innermost one. Type (2) bracts are more distal, oblong, acicular, long, with ciliate margins, spirally alternate, and each subtend a floral bud (Figs. 1a, b, 2.1, 4e).

Organogenesis, Elongation, and Differentiation—Floral meristems and bracts initiate acropetally along the inflorescence. The first floral meristems to initiate (the most basal ones) halt their development shortly after the initiation of the bracteole pair (Fig. 3a, b). The subsequent meristems, located closer to the apex of the inflorescence, rapidly reach the same developmental stage as the basal ones (Fig. 3a, b) and may even surpass them in the organogenesis phase (Fig. 3c). Consequently, the inflorescence exhibits a partially basipetal sequential developmental character, meaning the most developed buds, up to anthesis, are apical, despite their later initiation in the raceme. The basal buds, however, develop relatively quickly, with a short plastochrony between those and apical ones. Therefore, all buds in the inflorescence are at very similar developmental stages (Figs. 3b, c, 4e).

#### Flower

Organography—Flowers characterized by a long pedicel, approximately 3 cm long, pseudotubular, and orange-red. The bracteoles are fused for more than half their length, giving the flower a tubular appearance. The calyx has five sepals, appearing as four at anthesis due to the fusion of two adaxial ones, oblong sepals with an acute apex, the adaxial segment being slightly wider. The corolla has five equal petals, each approximately 1.5 cm long, clawed, elliptical, with an acute apex and slightly wavy margins. The petals are erect, opening slightly at the anthesis, while still overlapping. The androecium consists of a staminal sheath extending up to 1.5 cm long, formed by the conation of the filaments of eleven stamens, with dorsifixed, introrse anthers. The free portion of the filaments reaches up to 0.8 cm in length. The six antepetalous stamens have shorter filaments than the five antesepalous stamens. The sheath, with a villous inner wall, is discontinuous at the adaxial portion of the flower, creating a gap through which the ovary and style emerge (Fig. 2.2). The ovary is stipitate, with a tomentose surface, and contains 3–4 ovules. The stipe is adnate to the median adaxial portion of the hypanthium. The style is about 3 cm long and slightly curved to the abaxial direction, with a globose, papillary stigma. Petaloid stamens have been observed in rare pre-anthesis buds. In these cases, one petal is absent for each petaloid stamen; however, the total number of stamens in the androecium remains unchanged, counting the modified ones (Figs. 1a, b, m, 2.1–5).

Organogenesis, elongation, and differentiation—The first organs to form are the pair of bracteoles, with the position of the first one varying between the right and left sides of the meristem (Fig. 3a, b). Then, their margins meet, covering the floral meristem at the end of calyx initiation. Before this, the bracteoles press against the meristem, especially in the abaxial and median adaxial regions (Fig. 3d, f). The first sepal (S1) initiates in the median abaxial region, and the meristem acquires an obovate shape (Fig. 3e). The second sepal (S2), which forms in the adaxial region but more laterally, can sometimes be confused with the protuberance formed by the pressure of the bracteoles (Fig. 3f). The position of S2 varies between the right and left adaxial sides in different floral buds (Fig. 3h–l). The third sepal (S3) generally begins in the lateral position opposite S2, while the fourth sepal (S4) begins practically simultaneously with S3, on the opposite lateral side (Fig. 3h). The fifth and final sepal (S5) to begin is the second adaxial sepal, which begins fused with S2 congenitally (Fig. 3h–i). In the initial stages, they can be differentiated by their varying lengths and partially free apices (Fig. 3i, k, m). Thus, the calyx initiation pattern is helical and may occur in a clockwise or counterclockwise direction.

The five organs of the corolla initiate simultaneously (Fig. 3i, j), but they may develop at different rates during organogenesis (Fig. 3j–l). Generally, the adaxial petal elongates more slowly. After the corolla, the center of the meristem rises, revealing the carpel (Fig. 3j, k). The whorl of antesepalous stamens generally initiates with the simultaneous formation of five primordia (Fig. 3l), although one or two may become evident earlier than the others without a specific position. The initiation of antepetalous stamens could not be observed, but they begin forming after the external whorls are larger and cover them (Fig. 3m–o). The whorl of antepetalous stamens typically consists of six stamens, with two opposite stamens beginning at the adaxial position, between the carpel and the adaxial petal (Figs. 3p, 4a).

Later, the bud regains radial symmetry (Fig. 3s, t). The profuse elongation of the pedicel becomes more evident at later stages (Fig. 4e, f). The bracteoles elongate rapidly and cover the meristem. Their margins fuse after closing, besides the apex. As they elongate, the two adaxial sepals become indistinguishable from each other. The petals elongate similarly. During the differentiation process, the base of their blades tapers to form the claw (Fig. 3t). Subsequently, the antesepalous stamens differentiate into filament and anther, followed by the antepetalous stamens (Fig. 3r–t). The antepetalous stamens remain smaller than the antesepalous ones until anthesis (Fig. 4a). In late stages of differentiation when the stamens already have differentiated anthers, the bases of the filaments fuse through intercalary elongation, which will later form the staminal sheath (Fig. 4b). Fusion of the filaments does not occur between the pair of stamens opposite the adaxial petal, leading to the discontinuity of the staminal sheath at this point. The carpel begins to form the carpel cleft before the initiation of the antepetalous stamens (Fig. 3n–p). Prior to the differentiation of the anthers and filaments, the carpel cleft is almost closed, and the carpel acquires an elongated and laterally flattened shape (Fig. 3q, r). Its acute apex gradually elongates and curves abaxially to form the style (Figs. 3s, t, 4a). Concomitantly, the stigma differentiates into a papillary surface at the apex of the carpel (Fig. 4d). At the same time, trichomes form in the ovary region, beginning at the base (Fig. 4g). At more advanced stages, the style twists on itself and forms a spiral structure that elongates at anthesis (Fig. 4c, g). No evidence of the process leading to the formation of petaloid stamens was observed at early stages of development, nor was there any change in the standard number of initiated organs of the corolla and androecium. This is an uncommon but not rare phenomenon. Given that no evidence of this process was observed throughout the developmental study, it is possible that it expresses only in the late stage. Fig. 4h shows a petaloid stamen taken from a preanthesis bud.

### ***B. leucantha*** (Figs. 1c, d, m, 2.6–11, 5a–p, 6a–l)

#### Inflorescence

Organography—Simple, terminal, erect and/or lateral, capituliform, globose raceme. The flowers are congested and open acropetally, arranged like a pincushion and with alternate and spiral anthotaxy. There are two types of bracts: one protects the inflorescence from the base with distichous anthotaxy. They are ovate with a rounded apex and rigid, pubescent externally, overlapping each other and the innermost flowers, with the first 20–30 being sterile; type two bracts begin as type one, which gradually decreases in size, become spiralized and thinner until it becomes oblong, with an acute apex, flexible and covered in white trichomes. They occur in the most distal portions of the raceme. There are 100–120 flower buds per inflorescence (Figs. 1c, 2.6).

Organogenesis, elongation, and differentiation—Floral meristems and bracts have acropetal initiation and development. The meristems begin with a short plastochrony between them, so that with even more than 100 buds per inflorescence, few developmental stages can be observed in a single inflorescence (Figs. 5a–d, 6d). The inflorescence axis is thickened (Fig. 5d), and the buds are distributed over its entire surface. It is possible to observe the immediate proximity of the floral meristems during organogenesis in Fig. 5b, where only bracts and bracteoles were removed. This congested structure of the inflorescence has a profound impact on early stages of flower development. However, this impact is barely noticeable in later stages (Figs. 5c, 6d). Different meristems have different shapes and development rates depending on the pressure they suffer from nearby meristems. Meristems at the base of the inflorescence are wider and larger than meristems in the middle of the inflorescence, even though they are at equivalent stages (Figs. 5a, b, j, 6c, f). Flowers located in a same “whorl” reach anthesis almost simultaneously, organizing the inflorescence similarly to an Asteraceae’s head with rings of peripheral flowers all opening first and the flowers internal to the disk opening after (Fig. 1c).

#### Flower

Organography—Flowers ivory to slightly yellowish with bracteoles 4–4.5 cm long, fused up to about 3.5 cm in length, oblanceolate, apex acute, flexible, outer surface woolly. Calyx with five sepals, the two adaxial sepals fused, up to 2.2 cm long, oblong, apex acute, reflexed at anthesis. Corolla with five equal petals, 3.2 cm long, clawed, claw 0.4 cm long, elliptical blades, acute apex, with rigid base and central axis, and reflexed at anthesis. The androecium is composed of a staminal sheath formed by the filaments of eleven stamens, connate part up to 1.7 cm long, free part 4–6 cm long, without a pattern of variation among the stamens. The sheath is woolly internally, especially at the base, and discontinuous in the adaxial portion, leaving a gap through which the carpel curves slightly out of the sheath. The anthers are dorsifixed and introrse. The carpel is stipitate and the stipe is adnate to the adaxial wall of the hypanthium. The ovary is about 1.5–1.7 cm long, falcate in shape, laterally compressed, with a velvety surface, containing 5 ovules. The style is 6–7 cm long, curves slightly abaxially and ends in a globose and papillate stigma (Figs. 1d, m, 2.7–11).

Organogenesis, elongation, and differentiation—The first bracteole initiates on the left or right side of the meristem, and the second comes soon after (Fig. 5a, d). As they elongate, they exert pressure, especially on the medial abaxial and adaxial portions of the internal meristem (Fig. 5e, f). Before their margins meet, the first sepal (S1) initiates in the medial abaxial position (Fig. 5f). Due to the pressure exerted on the medial adaxial position, a protuberance is formed that can be confused with the second sepal (S2) (Fig. 5g). S2 initiates in the adaxial position, relatively lateralized and converging with the protuberance in the same position (Fig. 5g, h). Its initial position varies between the left or the right side of the adaxial portion. Next the lateral sepals arise (S3 and S4), whose initiation time is variable and may be simultaneous (Fig. 5h–j) or sequential (Fig. 5k, l). The fifth sepal (S5) is the second adaxial and begins fused with S2 (Fig. 5m). The initiation time of the last three sepals may occur before the initiation of the corolla (Fig. 5h–j) or after it (Fig. 5k, l). Fig. 5k and l represent the same meristem from two different angles to show that, although the corolla has already been initiated, S4 and S5 are not yet present. Although there is an erraticism due to variable modifications in different buds due to the pressure of the bracts, bracteoles, and other floral buds, there is a general helical pattern of initiation of the calyx.

Corolla initiation appears to be simultaneous, however, with development rates varying between organs and between meristems (Fig. 5j–o). In Fig. 5k and l, the abaxial petals appear larger, while in Fig. 5m, they all appear the same size, although with different shapes, and in Fig. 5n and o, the adaxial petals appear larger. Again, this erratic development between different buds appears to result from the differential pressure of each flower in the congested inflorescence. Furthermore, the calyx and corolla organs gradually become marginalized in the meristem as the inner whorls initiate (Figs. 5n, 6a). The antesepalous stamens, like the corolla, initiate almost simultaneously (Fig. 5n, o). However, both initially and later (Fig. 6h), they present different development rates. The whorl of antepetalous stamens has unidirectional initiation (Figs. 5p, 6a) and it has six stamens since two begin opposite the adaxial petal (Fig. 6a, b). Thus, the androecium normally contains eleven stamens in total.

Sometimes, a flower bud with ten stamens occurs (Fig. 6f). The carpel begins after the corolla and before the stamens (Fig. 5m, n). The bracteoles cover the bud between the end of calyx organogenesis and during corolla organogenesis. The sepals elongate slowly, and at the beginning of organ differentiation, they have not yet covered the internal whorls (Fig. 6f, g). The sepals elongate fused at the base (Fig. 6e). The adaxial petal is the first to differentiate with tapering of the apex, followed by the others (Fig. 6b, c). In general, all stamens elongate and differentiate equally (Fig. 6g). The stamens also develop equally, although the antepetalous remain smaller than the antesepalous (Fig. 6h, i).

At a much later differentiation stage, the bases of the filaments grow fused by intercalary growth (Fig. 6k). This process later forms a staminal sheath, which has a gap between the two adaxial antepetalous stamens, whose filaments do not fuse. The carpel begins to form its cleft after the end of organogenesis (Fig. 6c). After the beginning of the other whorls differentiation, its apex begins to elongate (Fig. 6g) and the style is slowly formed, which curves to the abaxial side (Fig. 6h, i), later forming a spiral (Fig. 6j).

### ***H. mimosoides*** (Figs. 1k, l, o, 2.12–18, 7a–t, 8a–h, 15d–f)

#### Inflorescence

Organography—Racemes are simple, terminal, axillary, or cauliflorous. The peduncle is short, erect, 2–6 cm long; the flowers have alternate spiral anthotaxy with short internodes. The bracts are about 1 cm long, elliptical, fibrous, green, and deciduous, with the proximal ones, at the base of the peduncle, being distichous and bearing no flowers, while the distal ones have spiral anthotaxy. There are about 6–8 flowers per inflorescence (Fig. 2.12).

Organogenesis and development—Floral meristems and bracts have acropetal sequential initiation and development. Each has a long plastochron that is more noticeable during late stages of differentiation, so that there is a large developmental interval between a floral bud and its adjacent ones (Figs. 7a, b, 8g). The peduncle elongates only slightly, so that even with long plastochrons between the buds, they are still very close to each other (Fig. 8g). There are few distinct stages of development within the same inflorescence.

#### Flower

Organography—The flowers have short pedicels about 0.5 cm long. The bracteoles are short, only 0.4 cm long, green, and are fused up to a little more than half their length. They are fibrous and green. The calyx is formed by five sepals, the adaxial pair being fused, about 3.5 cm long, oblong in shape, with an acute apex, purple with small white stripes at the base and a small green dot at the apex. The adaxial pair is slightly wider than the others and reflexed, while the other three sepals are erect on the abaxial side of the flower. The corolla is formed by three large and showy petals, the lateral ones and the adaxial one, while the two abaxial ones are reduced and hidden by the calyx at anthesis. The three petals are approximately the same size, with the adaxial petal slightly shorter and wider. They are spatulate, 6–6.3 cm × 2.8–3.1 cm, slightly wavy margin, and a truncated and emarginate apex and attenuated base. The adaxial petal has a ciliate margin at the base. The abaxial petal rudiments are ca. 0.3 cm long and filiform. The petals are purple, the adaxial one with conspicuous nectar guides in the form of white lines beginning from the base, ascending the central axis and spreading across the top half of the blade. The central line at the base is light pink, becoming cream towards the apex of the blade. The base of the other petals is light pink (Figs. 1k, l, o, 2.12–15).

The androecium has three stamens and six staminodes, all forming a bilateral and monadelphous sheath on the abaxial side, with filaments of varying length and conation and curving their free apices towards the adaxial side. The two lateral staminodes are 2 cm long, followed by two internal staminodes, with 4.5–5 cm long, followed by two internal stamens, with 6.5 cm long, then two internal staminodes with 5 cm long, and a central stamen, with 6 cm long. All or, sometimes only the four internal staminodes have antheroids. The height of the conation of filaments varies from 0.5 to 2.5 cm long. The sheath is pink and curves abaxially before the free filaments curve adaxially. The free portion of the five internal filaments have a few long and sparse trichomes. The anthers are dorsifixed, introrse and light pink (Fig. 2.16–17).

The carpel is stipitate; the pubescent stipe is partly adnate to the adaxial wall of the hypanthium, with the free part 0.9 cm. The light pink falcate ovary is 1.3 cm long, with white pubescence, curving slightly towards the abaxial side, and contains nine ovules. The pink style is 4.5 cm long, arches towards the abaxial side and then turns towards the adaxial side. It has an apical globose stigma with a papillary surface (Figs. 1k, l, 2.18).

Organogenesis, elongation, and differentiation—The undifferentiated floral meristem is laterally elongated (Fig. 7b). It expands and the first bracteole initiates on one lateral, followed by the second bracteole (Fig. 7b–d). They overlap first at the apex, leaving a small gap at the abaxial and medial adaxial positions before completely covering the meristem (Fig. 7d). They may form small projections at the adaxial medial position of the meristem where the bracteole margins meet (Fig. 7e, f). When these overlap, the first sepal (S1) initiates at the abaxial medial position (Fig. 7e). The second sepal (S2) initiates at the adaxial position, slightly displaced to the right or left of the meristem (Fig. 7f). The third sepal (S3) initiates on the side opposite to S2, quickly followed by the fourth sepal (S4) on the other side (Fig. 7g, h). The fifth sepal (S5) to initiate is the second adaxial that appears to be congenitally fused to S2 (Fig. 7i, j). Therefore, the calyx presents helical initiation.

The initiation pattern of the corolla is dubious, appearing to be a simultaneous initiation with developmental heterochrony from the beginning. However, we also consider the possibility of a unidirectional initiation, beginning with the abaxial pair, followed by the lateral pair and finally the adaxial one, with an extremely short plastochrony between the abaxial and lateral petals (Fig. 7k–n). The whorl of antesepalous stamens has unidirectional initiation, beginning with the abaxial, followed by the laterals, and finally the adaxial pair (Fig. 7m–o). The carpel begins shortly after the initiation of the petals and before the stamens (Fig. 7k, l). The bracteoles elongate and cover the floral meristem during organogenesis in the calyx (Fig. 7d).

However, during elongation, the calyx later surpasses the bracteoles and bursts through their cover (Fig. 8f). The calyx grows and covers the internal meristem during the organogenesis of the androecium. The petals, despite their simultaneous initiation (Fig. 7k), present an unequal rate of development (Fig. 7l–o). The abaxial petals elongate slowly and reach anthesis as rudiments (Figs. 7s, 8c). The lateral petals surpass the abaxial petals at the beginning of stamen differentiation (Fig. 7s). The adaxial petal is the slowest to elongate (Fig. 7l–n), but it also overtakes the abaxial ones at intermediate stages (Fig. 7s). During elongation it envelops the carpel dorsally and laterally, elongating between the lateral stamens (Figs. 7s, 8d).

The antesepalous whorl of stamens have unidirectional initiation, starting from the abaxial one (Fig. 7q, r). The lateral and abaxial stamens elongate similarly (Figs. 7q, 8a–c). The two adaxial stamens elongate more and slowly (Figs. 7t, 8a, b, e). At anthesis, these form the external pieces of the monoadelphous sheath and are the shortest stamens (Fig. 8e). The four antepetalous stamens elongate similarly and undergo differentiation but remain much smaller than the first three initiated (Fig. 8e). Furthermore, the elevation of the sheath occurs at a late stage by the fusion of the filaments by intercalary growth (Fig. 8e).

The carpel begins to form its cleft before the initiation of the antepetalous stamens (Fig. 7o, p). Its apex tapers and curves abaxially as it elongates to form the style, forming a spiral (Fig. 8a, b, d, h).

### ***M. suaveolens*** (Figs. 1i, j, n, 2.19–23, 9a–t, 10a–h)

#### Inflorescence

Organography—The laxly flowered racemes are simple, axillary or terminal, erect, the peduncle 6–10 cm long, with densely woolly indumentum, arranged in alternate spiral anthotaxis. One abaxial bract per flower is present, being ovate to elliptic, about 1 cm long, with pubescent outer surface and puberulent inner surface. Each raceme contains 15–25 flowers (Figs. 1i, j, 2.19).

Organogenesis, elongation, and differentiation—Floral meristems and bracts initiate and develop sequentially acropetally. The plastochrony of meristems are short but are relatively far apart so that the development of each is not greatly impacted by its neighbors (Fig. 9a–c). As the inflorescence elongates, the buds are organized spirally on the inflorescence axis. Bracts grow and differentiate very rapidly (Fig. 9c), where the apex of the inflorescence is still largely undifferentiated, except for the bracts.

#### Flower

Organography—The pair of bracteoles protects the flower in bud since the sepals are short and do not cover the inner whorls. The bracteoles are about 0.8 cm long, ovate, with a cuspidate apex, rigid, densely pubescent in the outer surface, and puberulent in the inner surface. The calyx has five small sepals, the adaxial pair slightly shorter. The sepals are triangular to ovate, with acute to acuminate apex, green, and about 0.1 to 0.2 cm long. The adaxial sepals may be free or partially fused from flower to flower. The corolla has one clawed, adaxial petal, white, with a 0.5 cm long claw and an orbicular blade, ca. 0.6 cm long, 0.7 cm wide, and with wavy margin. The lateral margins of the petal claw are slightly curved inward, forming a pseudotube, and its base is slightly auriculate, surrounding the stipe. The androecium is reduced to three reddish stamens that correspond to the abaxial and lateral antesepalous ones, rarely with a fourth stamen. It is common to see traces of aborted stamens. The filaments are free, ca. 1.8 cm long and pubescent at the base with a few long trichomes. The anthers are dorsifixed and versatile. The stipe is adnate to the adaxial wall of the hypanthium, villous on the apical surface and it curves towards the abaxial side of the flower. The ovary is trapezoidal in shape, laterally compressed, marginally villous and contains 1–2 ovules. The style is about 1.5 cm long with a globose apex and a papillary stigma. The hypanthium is gibbose and short (Figs. 1j, n, 2.19–23).

Organogenesis, elongation, and differentiation—The undifferentiated floral meristem is laterally elongated (Fig. 9c) but acquires a rhombic shape as it expands (Fig. 9g). The first bracteole varies in position between left and right laterally of the meristem, and the second begins shortly thereafter (Fig. 9c, d). The bracteoles thicken quickly, even as they begin to elongate (Fig. 9d–f). They overlap and cover the meristem (Fig. 9e, f), initially leaving a gap in the median adaxial position, by exerting physical pressure on the meristem (Fig. 9g). The shape of the still undifferentiated meristem is strongly influenced by the pressure of the bracteoles. Because of this, it is easy to confuse the abaxial and adaxial protuberances with already initiated sepal primordium (Fig. 9g).

The first sepal (S1) begins in the median abaxial position (Fig. 9h). The second sepal (S2) begins in the median adaxial position, opposite to S1 (Fig. 9i). The second and third sepals (S3 and S4) appear simultaneously on the sides, practically simultaneously with S2 (Fig. 9i). Fig. 9i shows that the four sepals have a very short plastochrony between them. It was not possible to observe the initiation of S5, which only becomes visible after the initiation of all the other organs (Fig. 9o). The initiation pattern of the calyx, therefore, is helical, however, with some level of erraticism.

The five petals initiate simultaneously, and the carpel initiates simultaneously with them (Fig. 9j). We also see an elevation abaxially in the antesepalous position. This probably corresponds to a stamen primordium (Fig. 9j). It was not possible to distinguish the moment of initiation of these three whorls, which can be considered practically simultaneous and superimposed given the very short plastochrony between them. In subsequent stages, we can barely observe the petals, which are suppressed as the other organs elongate (Fig. 9j–m). The lateral antesepalous stamens initiate shortly after the abaxial one (Fig. 9k–m). The adaxial portion of the meristem is slightly marginalized as the carpel and the first three stamens elongate, making it difficult to observe (Fig. 9k–n). The petals are visible during the intermediate stages (Fig. 9m–o).

Currently, we also see the two abaxial antepetalous stamen primordia (Fig. 9n–p). The last two stamens to initiate are opposite the lateral petal primordia, but it is uncertain whether they are of an antesepalous or an antepetalous nature (Fig. 9p–s). It is possible that one or both do not initiate or, if they do initiate, they are suppressed soon after, as seen in Fig. 9q, in which one of the lateral petals is also missing. Thus, the number of initiated stamens varies from 5–7 and the initiation pattern for both stamen whorls is unidirectional. In general, floral organogenesis presents a short plastochrony between each whorl and between the organs of the same whorl, as well as overlap in whorl initiation. On the other hand, there is a great heterochrony in organ development, observable from the early stages.

During the organ elongation, these are arranged at different heights of the meristem, with the abaxial ones higher than the adaxial ones (Fig. 9n–s). The bracteoles elongate and stiffen rapidly, and their margins fuse, before calyx initiation (Fig. 9f–g). Stomata are present on the inner surface of the bracteole during advanced stages of differentiation (Fig. 10h). The sepal primordia develop slowly and do not elongate enough to cover the inner whorls. The two adaxial sepals are the ones that take the longest to elongate, remaining slightly hidden under the adaxial petal primordium (Fig. 9l–s) which, although initially slow to elongate, is the only one of the corolla to elongate, differentiate and reach anthesis (Figs. 9s, t, 10a–d). It remains practically adjacent to the carpel from the beginning of its elongation until the point at which it exceeds it in length and envelops it from above and laterally, occupying the space between it and the stamens (Fig. 10a–d).

The other petals are visible as undifferentiated primordia during intermediate stages but are subsequently suppressed and are no longer visible at anthesis (Fig. 9p–s). The first three stamens to emerge are the only ones to develop, in most cases (Figs. 9t, 10a, e). Rarely does a fourth stamen develop and differentiate, reaching anthesis (Fig. 10c). This fourth stamen generally corresponds to one of the lateral antepetalous. As primordia, the stamens elongate rapidly, surpassing the corolla primordia and closely following the length of the carpel (Fig. 9l–p). Their differentiation occurs at the beginning of the formation of the carpel cleft (Fig. 9q–s). The remaining stamen primordia generally do not reach anthesis but are still observable at intermediate and late stages (Figs. 9r, 10b). The uneven shape of the floral meristem surface can be confusing as to the size of the primordia, so that the organs on the abaxial side, depending on the angle, appear to be much larger (Fig. 9n–p). Soon after initiating, the carpel takes up much of the central and adaxial space of the meristem, hiding the median adaxial region (Fig. 9l–p). Differentiation of the carpel cleft begins soon after all organs are formed (Fig. 9p, q). At later stages, the carpel tapers slightly at the base (Fig. 10b) and its apex curves almost perpendicularly to the abaxial side (Figs. 9t, 10a). The apex elongates to form the style, which gradually curves back on itself (Fig. 10f, g). Long trichomes form on the margins of the ovary (Fig. 10g).

### Paloue

The two species of *Paloue* here studied present similar organography and almost identical organogeny of their flowers. The main differences between species lay in their inflorescences and some details of flower organography. The differences in the organography and inflorescence development are punctuated in *P. speciosa* sections, but there’s only one description section of flower development for both species, with their differences being pointed out along the way.

### ***P. paraensis*** (Figs. 1g, h, p, 2.24–28, 11a–t, 12a–t, 15g–j) and ***P. speciosa*** (Figs. 1e, f, p, 2.29–32, 13a–t, 14a–h, 15k–m, p)

#### Inflorescence (*P. paraensis*)

Organography—Inflorescence is a simple, terminal, erect, globose, and capituliform raceme . The relatively short peduncle is about 8 cm long and bears 40–50 congested flowers subtended by cuculate bracts of slightly rigid and fibrous consistency and externally pubescent. The first bracts at the base of the peduncle have alternate distichous anthotaxy and are strongly overlapped, in addition to not subtending flowers. Later, they begin to spiral and bear flowers. The bracts of more distal buds generally overlap other more internal structures, contributing to the globose and compact appearance of the inflorescence. Generally, two to four flowers are open at a time in the same inflorescence. (Figs. 1g, 2.24).

Organogenesis and development—Floral meristems and bracts have acropetal sequential initiation and development. They present a relatively short plastochrony of initiation between floral buds, and it is possible to observe many stages of development in the same inflorescence. The flower buds are positioned very close to each other (Figs. 11a–c, 12h). In Fig. 11b, c, we see that the meristems tend to begin in a diametrically opposite position relative to the previous one in the peduncle. During development, as the axis of the inflorescence elongates and generates new meristems, the proximity between the flower buds increases and the inflorescence acquires a spiral anthotaxy (Fig. 12h).

#### Inflorescence (*P. speciosa*)

Organography—*P. speciosa* inflorescences differ from those of *P. paraensis* in having racemes with peduncles about 6–10 cm long, bearing 20–25 flower buds just slightly congested, not capituliform. The bracts are obovate and cuculate, and wine-colored. Generally one to two flowers are open at a time in the same inflorescence. (Figs. 1e, 2.29).

Organogenesis, elongation, and differentiation—Floral meristems initiate with a longer plastochron between them than those of *P. paraensis* (Fig. 13a-c). In addition, considerably more space is maintained between the flower buds (Fig. 13a).

#### Flower

Organography (*P. paraensis*)—The flowers have short pedicels up to 1.5 cm long and are arranged very close to each other on the peduncle, which contributes to their compactness. The bracteoles are about 2–2.5 cm long and are fused up to almost half their length. They are cuculate, surrounding the base of the flower, have a slightly rigid and fibrous consistency, a pubescent outer surface, and are greenish brown. The calyx has five white sepals (two fused adaxial ones), about 2 cm long, oblong to ovate, a rounded apex, and a villous inner surface, with the adaxial one being wider than the others (Fig. 1g, h, p).

The corolla has five petals, the lateral and abaxial ones being smaller, up to 2.5 cm long, and the adaxial one up to 3 cm long, oblong in shape, with a rounded to slightly emarginate apex, and a white color with a vinaceous base. The adaxial may present linear pink nectar guides at the base of the central region of the blade (Fig. 2.25). The androecium is composed of a short staminal ring 1 mm high, with three stamens corresponding to the abaxial and two lateral antesepalous, and six staminodes corresponding to the two adaxial antesepalous and two abaxial and two lateral antepetalous. The stamens are up to 5 cm long, while the staminodes are up to 2.5 cm long and are wine-colored. The filaments are free above the ring, villous at the base, and the anthers are dorsifixed and introrse. The staminodes do not form anthers (Fig. 2.26–27).

The carpel is stipitate, with the stipe adnate to the adaxial wall of the hypanthium and its apex curved toward the abaxial side. The ovary is trapezoidal and laterally compressed, about 1–1.2 cm long, with a velvety surface, light pink and bearing 10–11 ovules. The vinaceous style is about 4.5 cm long, ending in a white stigma with a papillose surface (Fig. 2.28).

Organography (*P. speciosa*)—The flowers are similar to *P. paraensis* but differ, primarily, by their smaller organs and by its reddish colors. The pedicels are up to 0.5 cm long. The bracteoles are about 1.2 cm long and wine-colored, besides being glabrous. The calyx has pink to red sepals up to 1.5 cm long. The petals are pink to red, oblong to elliptical, acute apex, with the adaxial one narrower and more withish, also surrounding the gynoecium even in anthesis, in addition to having a slightly auriculate base. The staminal ring is 0.1 cm high, with the stamens 2.5–3 cm long and staminodes ranging in size in the same flower from 0.8–1.2 cm long. The filaments are pink to red. The anthers are light pink. The ovary is 6–10 cm long, also light pink, and bears eight ovules. The style is 1.6–2 cm long and pink (Figs. 1f, p, 2.29–32).

Organogenesis, elongation, and differentiation (both *Paloue* species)—The first organs to emerge from the floral meristem are the two bracteoles, initiating one after the other, with the position of initiation of the first varying between left or right lateral (Figs. 11c–f, 13c–f). The bracteoles elongate more rapidly in length than in width, covering the apical surface of the meristem, while the median abaxial and adaxial regions are initially exposed (Figs. 11f, 13e). The first sepal (S1) initiates in the median abaxial position (Figs. 11g, 13g). The protuberance in the median adaxial region is formed by the mechanical pressure exerted by the margins of the bracteoles (Fig. 11f, h) and can be confused with the emergence of the second sepal (S2), but we observe that it initiates laterally in the adaxial region (Fig. 11i), varying, between meristems, between the right or left side (Figs. 11i–p, 13f–m). The second and third sepals (S3 and S4) initiate almost simultaneously in lateral positions, with a short plastochron, with S3 generally opposite S2 (Figs. 11j, 13h, i). In Fig. 13i, we see that a small gap remains between the bracteoles in the median adaxial position. The margin of the bracteole presses against the surface of S2, where we can see the mark left by it. The fifth sepal (S5) is the second adaxial and may be left or right depending on the position of S2 (Figs. 11k–p, 13j–m). Therefore, the initiation pattern of the calyx organs is helical.

The meristem internal to the calyx acquires a star shape where each of the vertices represents one of the corolla primordia that will initiate simultaneously (Figs. 11l, 13k, l), but have differential development. The adaxial petal takes longer than the others to elongate (Figs. 11p–t, 13o, q). After the corolla primordia initiates, we observe a central elevation in the meristem corresponding to the initiating carpel (Figs. 11m, n, 13e–m). The first three primordia of antesepalous stamens arise almost simultaneously in the lateral and median abaxial positions, opposite S1, S3 and S4 (Figs. 11p, 13n). At some moments, it is possible to observe elevations of a dubious nature in lateral positions that may correspond to the primordia of stamens (Fig. 11n, o). In *P. paraensis,* the two abaxial antepetalous primordia initiate before the two adaxial antesepalous, so that there is an overlap of the two whorls (Fig. 11q–s). The two adaxial antesepalous primordia and the lateral antepetalous primordia are observed at the beginning of their elongation (Fig. 11s, t). In *P. speciosa*, we see the unidirectional initiation of all five primordia of the antesepalous whorl 13o, p, but it was not possible to observe the initiation of the inner whorl of stamens, which only became visible when the other organs were more elongated (Fig. 13r). In both cases, no trace of initiation of the antepetalous adaxial stamen is observed. Thus, both cases present unidirectional initiation, although it is possible that the antesepalous whorl of *P. paraensis* presents bidirectional initiation, but with very short plastochrons between the first three primordia.

The first bracteole overlaps the second after their margins meet. They elongate vertically and fuse, except for the apical portion that remains free (Fig. 13f). The apical region of the larger bracteole acquires a domed shape as it covers the apical surface of the smaller one (Fig. 13f). The calyx elongates vertically with its base already fused (Figs. 11o, 13m). S2 and S5 gradually fuse their margins completely, becoming indistinguishable in anthesis. The corolla elongates slowly, but the petals differentiate rapidly with lateral widening of the lamina and tapering of the apex (Figs. 12d, q, 13q, s). We can see markings on petals made from the pressure exerted by the sepals (Fig. 11q, r). It is possible that the slower development of the adaxial petal is due to the pressure of S2 on it (Figs. 11p–t, 13s). When the adaxial petal reaches the same length as the carpel (Fig. 11t), its lateral margins grow to envelop it from above and laterally, through the space between it and the stamens (Figs. 12d, g, 14c). It surpasses it in length and begins to envelop it at the beginning of style growth (Fig. 12d). In *P. speciosa*, the base of the petal acquires a slightly auriculate shape that slightly invades the hypanthium cavity around the carpel (Fig. 14h). The other petals develop in a similar way to each other. The first three antesepalous stamens to emerge are just slightly larger than the others in the initial stages of elongation (Fig. 11s), but they quickly distance themselves in length and are the only ones to undergo the process of differentiation into anther and filament (Figs. 11t, 12a–g, 13t). Their differentiation produces an anther that is considerably larger in proportion to the filament (Figs. 12c–g, 14a–c). All three reach and follow the carpel in length and width. The other six stamens elongate slowly, without differentiating into anther and filament (Figs. 12a, e–g, 13t, 14b). In Fig. 14h, it is difficult to discern whether there is any structure corresponding to the antepetalous stamens, leading to the possibility that they are suppressed. The carpel takes up most of the center of the meristem soon after its initiation (Figs. 11p–s, 13m–p). The carpel cleft is formed during early stages of organ elongation (Figs. 11s, t, 13p). It deepens during staminal differentiation (Figs. 11t, 13s, t) and begins to close during style elongation (Fig. 12c). The style bends abaxially as it elongates (Fig. 14a–c), gradually forming a spiral (Figs. 12e, 14f, g).

## General comments

All studied species undergo the same fundamental process of hypanthium formation, though there are variations in their depth, shape, and other specific characteristics. The hypanthium generally begins to form during the later developmental stages through a vertical invagination of the meristematic tissue in the bud receptacle. This vertical elongation occurs specifically in the region between the carpel and the androecium. However, this process does not take place between the carpel and the median adaxial region. As a result, a stipe is formed that is adnate to the adaxial wall of the hypanthium (Figs. 4c, g, 6l, 10g, 12e, f, 14h). Table 2 presents the order of initiation and development of flowers and inflorescences in each of the analyzed taxa.

**Table 2:** Patterns of initiation and development of whorls of the studied species

	Inflorescence	Calyx	Corolla	Antesepalous st	Antepetalous st
*B. longipedicellata*	Partially basipetal	Helical	Simultaneous	Simultaneous	–
*B. leucantha*	Acropetal, nearly synchronous	Helical	Simultaneous	Simultaneous /erratic	Unidirectional
*H. mimosoides*	Acropetal, sequential	Helical	Simultaneous	Unidirectional	–
*M. suaveolens*	Acropetal, sequential	Unidirectional	Simultaneous	Unidirectional	Unidirectional
*P. paraensis*	Acropetal, sequential	Helical	Simultaneous	Unidirectional	Unidirectional
*P. speciosa*	Acropetal, sequential	Helical	Simultaneous	Unidirectional	–

– not observed, *st* stamens

## Nectariferous tissue of the hypanthium

All species exhibit structured nectariferous tissue divided into three distinct layers: the epidermis, nectariferous parenchyma, and subnectariferous parenchyma.

In *B. longipedicellata*, *P. paraensis*, and *P. speciosa*, the epidermis consists of elongated palisade cells (Fig. 15b, h, i, k). In contrast, *H. mimosoides* has small, rectangular epidermal cells (Fig. 15e). Stomata are concentrated in the basal region of the hypanthium wall, generally more abundant on the abaxial side, opposite to stipe (Fig. 15c, f, j, m).

The nectariferous parenchyma is composed of globoid cells with dense, intensely stained cytoplasm and generally thin cell walls. These traits are most pronounced in *P. paraensis*, and *P. speciosa* (Fig. 15g–i, k, l). In *H. mimosoides*, the cells have slightly thicker walls (Fig. 15e), while *B. longipedicellata* exhibits more rectangular cells (Fig. 15a, b). In *P. paraensis*, 1–2 layers of smaller and less stained cells occur just below the epidermis, differentiating them from the underlying nectar parenchyma (Fig. 15g–i).

The subnectariferous parenchyma varies greatly among species. *P. paraensis* has a prominent subnectariferous parenchyma composed of globoid, misshapen cells with thicker walls and less intense staining (Fig. 15h, i). Marginal vascular bundles are present but are not closely associated with the nectar parenchyma (Fig. 15g). *P. speciosa* exhibits damaged subnectariferous parenchyma in some samples, making visualization difficult. However, larger, less intensely stained, rectangular cells can be observed, along with a vascular bundle marginal to the nectar parenchyma (Fig. 15k). *B. longipedicellata* has larger subnectariferous parenchyma cells, which are slightly less stained and intersected by a vascular bundle (Fig. 15a, b). *In H. mimosoides*, the subnectariferous parenchyma was not observable.

Idioblasts were observed in all species, distributed primarily along the bracteoles and sepals (Fig. 15d, n, p). These cells are very large and intensely purple-stained, likely indicating the presence of mucilage. Those tissues were also stained with Toluidine Blue.

In *B. longipedicellata* (Fig. 15o), the abaxial wall of the hypanthium is covered with what appears to be modified trichomes. However, these structures were not observed in the anatomical sections or other buds, such as the one shown in Fig. 15c.

## Discussion

*General patterns—*Some of the common features of Detarioideae flowers that were observed in most of the studied species here are the staminal ring, which originates from the fusion of filament bases, which can, in some cases, extend to form sheaths or tubes (Bruneau et al. 2014; Cowan and Polhill 1981a; Tucker 2000b); the style, curving toward the abaxial side as it elongates during floral development (Prenner and Cardoso 2017; Tucker 2000a, 2000b, 2000c, 2001a, 2001b, 2002a, 2002b, 2003c); and the presence of two conspicuous and persistent bracteoles (Cowan and Polhill 1981a, 1981b; Tucker 2000a). 

The initiation of whorls in the meristem of the analyzed taxa follows a mixed acropetal pattern, beginning with the calyx, followed by the corolla, carpel, the whorl of antesepalous stamens, and finally the whorl of antepetalous stamens. This pattern, where the carpel is not the last organ to form, is typical of nearly all Fabaceae (Tucker 1987).

In *M. suaveolens*, the initiation of the corolla, carpel, and antesepalous stamens occurs in close succession, almost simultaneously. For *H. mimosoides*, the initiation of the carpel occurs alongside the corolla. *B. leucantha* occasionally shows overlapping initiation of the calyx and corolla, while in *P. paraensis*, overlap occurs between the two stamen whorls.

*Development and morphology of inflorescences and bracts—*The inflorescences in the taxa analyzed here consist of the same basic structure: racemes with spiral anthotaxy and, at least initially, acropetal sequential initiation. Some factors that play an important role in generating morphological diversity are: 1) relative length of the peduncle; 2) number and morphology of bracts; 3) number of flowers per inflorescence; 4) differences in plastochrony between the emergence of different floral meristems; 5) size of the pedicel and/or hypanthium; 6) relative size of the flowers. In table 3, some of these parameters are compared*.*

**Table 3:** Morphological diversity of the inflorescence of the studied species

Species	Inflorescence axis	Flowers per inflorescence	Plastochron between flowers	Pedicel/Hypanthium	Flower size
*B. longipedicellata*	Short	8–10	Short	Long pedicel; medium hypanthium	Large
*B. leucantha*	Short and robust	100–120	Short	Short pedicel; medium hypanthium	Large
*H. mimosoides*	Short	6–8	Long	Short pedicel; long hypanthium	Large
*M. suaveolens*	Medium	15–20	Medium	Both short	Small
*P. paraensis*	Short and robust	35–40	Short	Both short	Large
*P. speciosa*	Long	20–25	Long	Short pedicel; medium hypanthium	Medium

Most species of *Brownea* and *Browneopsis*, as well as some of *Paloue*, such as *P. paraensis*, have capituliform racemes, which are characterized by many congested flowers with a relatively short and thick peduncle and with reduced internodes between flowers (Arroyo 1981; Klitgaard 1991; Redden et al. 2018). Capituliform racemes, although common in the Brownea clade, are uncommon in Detarioideae (Bruneau et al. 2014; Cowan and Polhill 1981a, 1981b), but can be found in some species of the genus *Cynometra* L., formerly included in *Maniltoa* Scheff. (Lewis et al. 2005; Radosavljevic 2019). It is possible that, in the context of the Brownea clade, this type of capituliform and/or very congested inflorescence is an apomorphy for the clade that unites *Brownea* and *Browneopsis*, as well as for some lineages within *Paloue*, and that lax and few flowered inflorescences in a few species like *B. longipedicellata* are cases of reversal of this character. A study of character evolution is needed to evaluate these hypotheses.

The structure of this inflorescence is achieved through the development of numerous floral meristems with short plastochrony between them, which develop on a central axis with reduced internodes and with short pedicels. *B. longipedicellata*, however, has non-capituliform and lax racemes with few flowers (generally 7–10). The floral buds, although with short plastochrony between them, are few and have long pedicels. Besides that, *B. longipedicellata* presents basipetal sequential development, which may be due to greater mechanical pressure exerted by the bracts at the base of the inflorescence. Bull–Hereñu et al. (2022) commented on such development in some species of Araceae, Asteraceae, among others, in which the most basal meristems present a delayed development due to the pressure of specialized protective organs such as involucres and spathes. The case of *B. latifolia* (Tucker 2000b), whose meristems still in early stages synchronize their development, is also rare in Detarioideae and uncommon in Fabaceae in general, excepting the tribe Mimoseae (Caesalpinioideae) (Tucker 1987). In Detarioideae, Tucker (2002b) also recorded initially synchronous development for *Berlinia grandiflora* (Vahl) Hutch. & Dalziel, and basipetal development in *Paramacrolobium coeruleum* (Taub.) J.Léonard. However, there are no images of these records.

In inflorescences of *B. leucantha*, numerous meristems with short plastochrony and close to each other lead to intense mechanical pressures, evidenced by meristems at the base of the inflorescence (Figs. 5j, 6c, f) that are wider than meristems in the middle of the inflorescence, yet at the same developmental stage (Figs. 5i, 6b, e). In addition, the erraticity, especially of the elongation of the whorls and the variable shape of adjacent meristems at very similar stages, are some examples of the impact generated by those pressures (Bull–Hereñu et al. 2022; Endress 2006). Comparing young inflorescences (Fig. 5a, b) to developed ones (Figs. 5c, 6d), we see that the competition for space generates greater impact during early stages of organogenesis. It is possible to trace several similarities between the development of the inflorescence of *Brownea* with genera of the Mimoseae tribe in the subfamily Caesalpinioideae, such as *Inga*, *Parkia*, and others, whose inflorescences are commonly heads, glomerules or capituliform racemes, congested, with numerous flowers, simultaneous or almost simultaneous development of one or more whorls (generally the corolla and androecium), and radially symmetrical flowers (Paulino et al. 2016; Pedersoli and Teixeira 2016; Prenner 2004a; Ramirez-Domenech and Tucker 1990; Tucker 1987). Furthermore, Arroyo (1981) highlights species of *Parkia* and *Inga* with chiropterophilous flowers, a pollination syndrome also cited for *Browneopsis* (Knudsen and Klitgaard 1998). Many of these floral architectural patterns are also seen in the flowers of some *Brownea* differing mainly in the flowers presenting pseudotubes, while *B. leucantha*, has a reflexed corolla and a shallow staminal ring (Arroyo 1981; Bruneau et al. 2014; Klitgaard 1991), highlighting that *B. longipedicellata* is an exception to most of these character states found in most of its genus. In *Brownea, Browneopsis* and in many Mimoseae genera, the inflorescence acts as an attractive unit structure for the pollinator (Arroyo 1981) and that the erraticity observed in the organogenetic patterns of *Brownea* is also present in some of the Mimoseae genera with similar inflorescences, such as *Senegalia* and *Vachellia* (Alvarado-Reyes et al. 2024; Gomez-Acevedo et al. 2007; Gomez-Acevedo 2021), highlighting the possibility that the impact of this inflorescence structuration is one of the factors acting on floral ontogeny.

*P. paraensis* and *P. speciosa* diverge in their inflorescence development. While *P. speciosa* has a loose raceme with few flowers, *P. paraensis* has a congested raceme with many more flowers. The divergence between the pathways that led to these is due to the different plastochrony between the emergence of the floral meristems of each species, longer in *P. speciosa* and shorter in *P. paraensis*. This may be one of the main factors enabling the formation of so many flowers in *P. paraensis* (Figs. 11a, b, 12h), while in *P. speciosa*, the long plastochrony causes more basal flowers to already be very developed (Fig. 13a, b) when apical flowers appear, which raises questions about mechanical forces and the distribution of resources along the inflorescence. Larger and heavier inflorescences with many flowers have a greater need for resource input, which may explain the thin and soft peduncles of *P. speciosa* and the thick and rigid ones of *P. paraensis*. Despite the great difference in the number of flowers, these two species, as well as in another similar species (*P. princeps*), have few flowers open per inflorescence at the same time, contrasting with *B. leucantha*, where most of the flowers can be in anthesis simultaneously, forming sometimes a peripheric ring of flowers (Fig. 1a). In other *Brownea* species, all flowers in the inflorescence can be at anthesis simultaneously. This characteristic is, however, uncommon in *Paloue*, since some species like *Paloue durissima* (Ducke) Redden have short inflorescences with about 5-10 flowers, like those of *P. speciosa*, but about half of the flowers are in anthesis simultaneously and other species like *Paloue guianensis* Aubl. have strongly reduced inflorescences with generally a single flower open at a time (Redden et al. 2018; Rodrigues et al. personal observation). The main developmental differences in floral morphology between the *P. paraensis* and *P. speciosa* occur at late stages, while organogenesis and elongation are similar, which is usually observed among closely related taxa (Tucker 1984, 1997), with exceptions (Bruneau et al. 2014). We observed that differences in the development of the inflorescence, prior to organogenesis, also play an important role in the differentiation of these structures in closely related taxa. This phenomenon can also be observed between *B. longipedicellata* (present study) and *B. latifolia* (Tucker 2000b). *H. mimosoides*, like *B. longipedicellata*, has racemes with short internodes and a few flowers with large plastochrons between them. *M. suaveolens* presents a typical raceme with intermediate characteristics concerning the five factors of morphological variability in Table 3. It is possible to see how the recombination of these factors generate morphologically variable inflorescences without necessarily changing the basic structure of the raceme.

Another feature found in the development of the inflorescences of all species of the clade studied here (except *M. suaveolens*) is the presence of sterile bracts with a distichous arrangement at the base of the inflorescence. From a certain level on the main axis of the inflorescence, these gradually begin to support floral buds and become spiral, a pattern common to the majority of species of Detarioideae and all of Fabaceae, with rare distichous exceptions in Detarioideae, with records for 10 genera, including, for example, *Sindora* Miq., *Goniorrhachis* Taub., *Copaifera* L. and *Cynometra* (Dwyer 1951; Maior et al. in prep; Radosavljevic 2019; Tucker 1987, 2003c). In Fabaceae, fully distichous inflorescences are a synapomorphy of the subfamily Dialioideae (Falcão et al. 2020, 2023) and are present in a few genera of Papilionoideae (Monteiro et al. in prep; Prenner 2013; Tucker 1987). The distichy present in the basal portions of the inflorescences of taxa of the Brownea clade may also be related to a continuity of the pattern observed for their leaves, since these species have strongly distichous phyllotaxis (Klitgaard 1991; Redden et al. 2018; present work). Despite the possibility that these partially distichous bracts, found in almost all studied taxa of the Brownea clade, are a synapomorphy for this group, studies focused on the origin of the inflorescence are necessary for other lineages of Detarioideae since such stages are commonly not treated in ontogenetic studies. Here we found the greater expression of this character in species with congested capituliform inflorescences (*B. leucantha* and *P. paraensis*) and in one species with lax racemes (*B. longipedicellata*), where the basal, sterile and distichous bracts are larger in relation to the inflorescence and, apparently, more numerous. However, of the closest relatives of *B. longipedicellata,* most have capituliform inflorescences and, despite the need for studies to reconstruct ancestral character states, it can be conjectured that the lax inflorescences with few flowers of *B. longipedicellata* represent a reduction from the ancestral capituliform character, while the original bracts are still present. Furthermore, more than one bract morphotype may occur in the same raceme, as is the case of *B. longipedicellata* and *B. leucantha*.

In all taxa studied, the initiation of the first bracteole varies in position in relation to the right or left side of the meristem. This generates a cascade effect that leads to the development of flowers where the organogenesis of the calyx also occurs in a mirrored manner, with flowers where the helicoid pattern occurs in a clockwise direction, with the second sepal (S2) to form being the left adaxial one, and other flowers where the direction is counterclockwise and the second sepal to form is the right adaxial one. Consequently, sepals three (S3), four (S4) and five (S5) will follow these patterns. The first sepal (S1) is the only one that is never affected, always appearing in the median abaxial position. Such variations may also extend to the corolla, allowing different imbrication patterns at anthesis. In this study, this phenomenon was observed in all species except *M. suaveolens*. In general, the variation in position occurs between meristems of the same inflorescence, except in *P. speciosa*, in which we observed that the variation occurs only between meristems of different inflorescences. The variation that occurs with the first bracteole is possibly caused by pressure on one side of the meristem by a bract further up the inflorescence axis (Figs. 11b, c, 13c), but this can only be seen more clearly in the case of *P. paraensis* and *P. speciosa*, while in the others the cause is not so clear.

In the case of sepals, we observed that, in most species, S2 begins opposite the largest/first-to-emerge bracteole; however, in *P. speciosa*, where all meristems of a given inflorescence present S2 in the same position, this pattern is broken. One possible explanation is that, at the time of S2 initiation, the largest bracteole is further away from the surface of the meristem because it is already overlapping the smaller bracteole. The latter, in turn, is closer to the meristem and, therefore, causes greater pressure on the side on which it is located (Fig. 13e, f). *H. mimosoides* and *B. longipedicellata* have few floral stages in the same inflorescence, making this comparative ontogeny of sepals difficult. However, the inflorescences of *P. paraensis* and *B. leucantha* have numerous buds in different stages, which generates many variables of pressure, making it difficult to find a pattern in the variation of the position of the bracteoles and sepals of the meristems. In *M. suaveolens*, S2 begins in the median adaxial position and S5 forms in much later stages, and both take a long time to elongate compared to the other sepals of the bud, which makes their observation difficult. Other studies mention this variation between the right and left sides in cases of helical calyx initiation pattern (Prenner 2004b; Prenner and Cardoso 2017; Tucker 1984, 2000a, 2000b, 2001a), however, without delving into the cause of such variations. Particularly in the subfamily Dialioideae, this effect mirrored in the development of bracteoles and perianth is present in several taxa, extending, in the genus *Dicorynia*, to the androecium and gynoecium and culminating in enantiostyly during anthesis (Falcão et al. 2023). Similar cases are possibly present in the tribe Cassieae of subfamily Caesalpinioideae (Marazzi and Endress 2008; Tucker 1996)

*Bracteoles, reduction events, and evolutionary parallelism in Detarioideae*—Partially fused bracteoles are considered a synapomorphy for the Brownea clade. This character state is present in most taxa of the clade, with reversal only in *Heterostemon ingifolius* Sandwith and most *Macrolobium* species, whose bracteoles open fully at anthesis (Bruneau et al. 2014; Cowan 1976; Redden et al. 2010; Tucker 2000b). In *Macrolobium* section *Stenosolen*, there is partial fusion of the bracteoles only on the adaxial side of the flower (Cowan 1953). Both cases of reversal may have occurred through different pathways of floral development. In *H. ingifolius*, it is possible that the loss of this characteristic occurred due to the reduction of the bracteoles, which are free and deciduous (Cowan 1976). On the other hand, in the case of *Macrolobium*, it is possible that this loss occurred through the enlargement and thickening of the bracteoles and its dominance as the protective organs of the flower, which, in many cases present strongly reduced sepals (Cowan 1953). In the floral development of *M. suaveolens*, this thickening process precedes the fusion process observed in the other taxa studied.

Tucker (2000a, 2000b, 2000c, 2001a, 2001b, 2002a, 2002b, 2003b, 2003c), in her studies of floral development for the current Detarioideae, divided the taxa of the subfamily into two groups based on the shape of the floral meristem at a stage prior to corolla initiation and after the beginning of bracteolar elongation: the circular and the omega types. The circular ones are species whose meristem is circular and paired with relatively small bracteoles, while the omega presents meristem dorsoventrally flattened with bracteoles already large during the organogenesis of the calyx. In the analysis by Bruneau et al. (2014), it was established that not all taxa with large bracteoles at the beginning of floral development are of the omega type, but that all of the omega type have large and thick bracteoles. Tucker studied several genera considered to be in the omega group from the tribe Amherstieae, Afzelieae and Detarieae (Tucker 2000a, 2002a, 2002b, 2003c). The images of the floral development of *Sindora klaineana* Pierre ex Pellegr. make it unclear whether the author would place it in the omega group (Tucker 2003c), as the few images available of this stage suggest the taxon would be better positioned in the circular group (present study). Several studies indicate the division between omega and circular groups is an artificial circumscription (Bruneau et al. 2000, 2014; Estrella et al. 2018), however, they draw attention to the occurrence of several lineages in Detarioideae, especially in the Amherstieae tribe, in which there was a profuse elongation and thickening of the bracteoles that generated different consequences for floral development.

Among all Detarioideae species that were ontogenetically addressed to date, it is possible to classify 19 species as omega-type and 19 as circular-type. *Saraca* L., from the Saraceae tribe, is not included in this assemblage because, although it is considered part of the circular group, its species ontogenetically addressed present particularities of floral development that do not apply to the variables being analyzed here (Tucker 2000c). *Goniorrhachis marginata* Taub., *Colophospermum mopane* (J. Kirk ex Benth.) J. Léonard, *Copaifera langsdorfii* Desf., *Hymenaea verrucosa* Gaertn. and *Brodriguesia santosii* R.S. Cowan were classified as belonging to the circular group based on our observations of data made available by other studies (Citeli et al. in prep; Kochanovski et al. 2018; Krüger et al. 1999; Pedersoli et al. 2010; Prenner and Cardoso 2017).

Comparing the two groups regarding corolla reduction, the omega group has 79% of cases of reduction (15 of 19 species), while the circular group has 42% of cases (eight of 19). On the other hand, androecium reduction is practically as frequent in the circular group (four of 19 (21%)) as in the omega group (six of 19 (31,5%)). The calyx, although showing few cases of reduction in numbers among Detarioideae, notably in *Colophospermum* J. Léonard*, Aphanocalyx* Oliv., and *Bikinia* Wieringa, presents several cases of reduction in size and shape during late stages of development until anthesis, especially in the omega group, as in *Gilbertiodendron klainei* (Pierre ex Pellegr.) J. Léonard, *Tetraberlinia tubmaniana* J. Léonard, *Microberlinia brazzavillensis* A. Chev., *Brachystegia tamarindoides* Welw. ex Benth., among others (Tucker 2000a, 2002a, 2002b). These data can be checked in Table S1 and Table S2 available in the Supplementary Information. Similarly, petals are often reduced in size and shape, supporting the idea that, in Detarioideae, the reduction is, in its vast majority, due to suppression or interruption of growth events in intermediate to late stages and, rarely, due to absence since initiation or suppression in early stages of development. This contrasts, for example, with the frequent reduction events observed in the subfamily Dialioideae, which are, in the majority, due to suppression immediately after emergence, in early stages of development or, more rarely, absence since initiation (Falcão et al. 2020, 2023; Zimmerman et al. 2013). It is important to emphasize that these observations correspond only to the scope of ontogenetic studies of Detarioideae, therefore, they do not contemplate the complete morphological diversity of flowers in the subfamily, in addition to being subject to a bias in the choice of taxa by the authors.

Comparing the floral development of *M. suaveolens* with that of other omega-type taxa, it is possible to trace significant similarities, especially at the beginning of organogenesis, such as the shape of the meristem and bracteoles, the overlapping initiation of whorls, reduction events that involve the non-initiation of organs or organs that initiate but are later suppressed and/or reduced at anthesis (Tucker 2000a, 2002a, 2002b, 2003c). *M. suavolens*, during organogenesis, especially resembles *Aphanocalyx djumaensis* (D.Wild) J.Léonard (Tucker 2000a). However, *Macrolobium* is not closely related with any of the other omega-group taxa (Bruneau et al. 2000, 2001; Estrella et al. 2018).

The pronounced development of bracteoles soon after their emergence and its relationship with reduction events in Detarioideae have been previously discussed (Bull–Hereñu et al. 2022; Tucker 2000a), as well as other variations in organogenesis caused by changes in the shape or presence/absence of bracteoles have already been identified for other groups of Fabaceae (Prenner 2004b). However, these reduction events occur more frequently in the perianth and androecium reduction events are possibly more frequently motivated by other causes, not related to the bracteoles. In addition, some cases of extreme corolla reduction such as *Crudia* Schreb., *Copaifera* and *Colophospermum* also involve different ontogenetic processes (Kruger et al. 1999; Tucker 2001a; Pedersoli et al. 2010). Finally, despite the ontogenetic similarities between the different lineages of the omega group, they still retain numerous differences, evidencing the analogy of these processes. The main point in common is the pair of highly developed bracteoles, so that the reduction events are possibly a consequence of this.

*Corolla and androecium—*Tucker (1988, 1989a, 2000a, 2000d) discusses various forms of meristic variation that can occur during floral development, including the fusion of organs, organ abortion, and non-initiation of organs (Tucker 2000a, 2001a), which plays a critical role in floral merism, especially as it can alter the organogenesis of subsequent floral whorls (Ronse de Craene 2016; Tucker 1988, 1989a, 2000d). In the corolla and androecium of Detarioideae taxa, reduction commonly occurs through the abortion of initiated organs. However, some organs may remain as vestigial structures at anthesis. These reductions often result in great variation of floral symmetry within a genus, as in *Paloue*, like *Paloue brasiliensis* Ducke and *Paloue coccinea* (M. R. Schomb. ex Benth.) Redden (Redden et al. 2018). Among the Brownea clade, the main differences in flower morphology are related to the corolla and androecium. Table 2 shows all the species analyzed with simultaneous corolla initiation, but with different rates of development immediately after organogenesis. In general, the adaxial petal initially elongates more slowly, but later equalizes and sometimes surpasses the others in length, as we can see in floral development of *H. mimosoides* and *M. suaveolens* (Figs. 7l-o, 9j, l-n). This is a common process in several Detarioideae taxa, such as *Hymenaea* L. (Kochanovski et al. 2018), *Amherstia* Wall., *Crudia*, *Schotia* Jacq., *Neochevalierodendron* J. Léonard (Tucker 2000b, 2001a, 2001b, 2002b) and *Brodriguesia* R.S. Cowan (Citeli et al. in prep), and is possibly due to the pressure exerted by the two early fused adaxial sepals. After their elongation, the pressure reduces, creating space for the petal to develop. In some of the taxa on which this process occurs, such as in three *Schotia* species (Tucker 2001b) and *Neochevalierodendron stephanii* (A.Chev.) J.Léonard (Tucker 2002b), the corolla is indicated as having unidirectional initiation, however, we could observe in the images provided in these studies that these are cases of simultaneous initiation, but with different rates of development shortly after the organs appear.

The differential elongation of the corolla that is in fact reflected in the flower in anthesis occurs at later stages, through processes of heterochrony of the primordia, such as we see in *H. mimosoides*. The development of *M. suaveolens* involves processes of heterochrony not only between organs of the same whorl, but between whorls, and it is possible to observe the impact that the more accelerated elongation of the abaxial and lateral antesepalous stamens has on the elongation of the petals (Fig. 9l–n).

In Fabaceae, initiation of a whorl generally begins after initiation of the previous one has finished (with the exception of the carpel), however, overlapping initiation of two or more whorls is not uncommon (Bruneau et al. 2014; Tucker 1989b, 2003b). In *P. paraensis*, the two stamen whorls overlap (Fig. 11q, r), possibly due to the long plastochrony between the primordia of the antesepalous whorl. This type of overlap is common in Papilionoideae, where unidirectional whorl initiation predominates (Tucker 1989b). On the other hand, in *M. suaveolens*, as well as in *Brachystegia*, *Julbernardia* Pellegr., *Bikinia*, *Aphanocalyx*, among others of the omega group (Tucker 2000a, 2003c), the overlapping of the corolla with the androecium is probably caused by the impact of the pressure of the bracteoles on organogenesis, which shortens the available space in the floral meristem and delays the initiation of the calyx and corolla, so that they overlap the following whorls (Tucker 2000d, 2002a). Tucker (2000a, 2002a) related this overlapping phenomenon to the emergence of a meristematic ring in the androecium whorl of *A. djumaensis*, *Bikinia durandii* (F. Hallé & Normand) Wieringa, *Microberlinia bisulcata* A. Chev. and *B. boehmii* Taub. However, the structure that the author identifies as a meristematic ring is quite different from those that occur in *C. mopane* (Krüger et al. 1999) or in the tribe Swartzieae of Papilionoideae (Paulino et al. 2013; Tucker 1990, 2003a) and Mimoseae of Caesalpinioideae (Alvarado-Reyes et al. 2024; Paulino et al. 2016; Prenner 2011), in which the meristematic ring is related to the expansion of a region, which often results in staminal proliferation and, more rarely, reductions in the corolla. Such an exception is the case of *Ateleia* (DC.) D.Dietr., of the tribe Swartzieae, which presents a meristematic ring, but, in general, without staminal proliferation and some cases of reduction (Tucker 1990). In the case of *Aphanocalyx*, *Bikinia*, *Microberlinia* and *Brachystegia* Benth., in which Tucker (2000a, 2002a) suggested the occurrence of a meristematic ring, there was, on the contrary, a restriction of space in the meristem with consequent overlapping of whorls and without alteration in the standard number of ten stamens. It is possible that, in these cases, the overlapping whorls, combined with the shortened plastochrony between the organs, as well as the delayed elongation of the outer whorls, generated a ring-shaped conformation around the carpel like a meristematic ring that, however, has different causes and implications for floral development.

The reduction of the androecium from the standard number of ten to three stamens at anthesis is a process that occurs very similarly in *M. suaveolens, H. mimosoides, P. paraensis* and *P. speciosa*. In all, three of the nine initiated stamens become fertile and the other six become staminodes, except for *M. suaveolens* which does not have any staminodes at anthesis. Among the six staminodes of *H. mimosoides*, four of them (abaxial and lateral antepetalous) have developed anthers, but they are smaller than those of the three larger stamens. These two structures are considered staminodes in the literature (Cowan 1976) but studies about their fertility have not yet been carried out, so the nature of these four intermediate organs is not entirely certain.

It is possible that the reduction from ten to nine initiated stamens is a plesiomorphic characteristic, not only for the clade comprising the genera *Heterostemon, Macrolobium*, and *Paloue*, but also for the Brownea clade, given the occurrence of this character state for the sister genera *Brachycylix* and *Ecuadendron*, as well as in other genera outside the Brownea clade, but closely related phylogenetically, such as *Amherstia* and *Tamarindus* Tourn. ex L. (Tucker 2000b, 2002b). Considering the character state of nine stamens as synapomorphic or even plesiomorphic for the Brownea clade, it would imply that there has been a reversal in the clade that includes *Brownea* and *Browneopsis*, whose species generally have ten or more stamens. Considering this character state as homoplastic, it would have occurred two times in the group: 1) in *Ecuadendron + Brachycylis*; and 2) in *Macrolobium + Heterostemon* + *Paloue*. Besides the reduction from ten to nine initiated stamens, all species of *Macrolobium* and *Heterostemon* and nine of the 16 species of *Paloue* present only three developed stamens and a variable number of staminodes at anthesis. Within *Paloue,* there are two morphologically distinct groups of species which mostly appears as relatively supported clades in some phylogenies, one with three stamens and six staminodes, and other with nine stamens (Redden et al. 2018). If we consider the three stamens as synapomorphic for the trio of genera, there would be a reversion in the *Paloue* group with nine stamens, except in *Paloue emarginata* (R.S. Cowan) Redden, which is positioned in this group, but has only three stamens and six staminodes (Redden et al. 2010, 2018) or such reduction is homoplastic, occurring several times within the clade. Studies on the evolutionary reconstruction of ancestral character states are therefore necessary.

The extra stamen of *B. longipedicellata* and *B. leucantha* always occurs in the adaxial region between the adaxial petal and the carpel (Figs. 3p, 6a, b). In *B. latifolia*, the position of the extra stamen varies (Tucker 2000b). In the case of taxa with a reduction from ten to nine stamens, the missing stamen always corresponds to this same position, according to current ontogenetic data (present study; Tucker 2000b, 2002b). More cases need to be evaluated, especially concerning *Browneopsis*, *Brachycylix* and *Ecuadendron*, which have not been addressed ontogenetically, such as *Browneopsis disepala* (Little) Klitgaard, which presents staminal proliferation of up to 26 stamens (Klitgaard 1991), to better understand this process and why this specific region of the meristem appears to present greater lability. One possibility is that the dorsoventrally flattened shape of the floral meristem in the adaxial region of the flowers of *B. longipedicellata* and *B. leucantha* (Figs. 3n–p, 6a–c) provides more space and may facilitate the emergence of extra organs (Claßen-Bockhoff and Meyer 2016).

In taxa on which only nine stamens are initiated, the adaxial antepetalous region that would normally be occupied by a stamen is occupied by the expanding carpel, so that it is close to the adaxial petal during elongation. We see this in *H. mimosoides*, *M. suaveolens*, *P. paraensis* and *P. speciosa* (Figs. 7s, 9s, 12b, 13s), as well as in *Amherstia nobilis* Wall., *Tamarindus indica* L. and *Afzelia quanzensis* Welw. (Tucker 2000b, 2002b). Tucker (2002b) states that the *Gilbertiodendron* species analyzed ontogenetically had ten initiated stamens, but from the images it was only possible to observe nine, the missing one corresponding to the adaxial antepetalous region. In addition, there is also an approximation between the carpel and the adaxial petal. *P. coeruleum* has nine initiated stamens; however, it was not possible to verify this characteristic, since no images were available. In taxa outside the Brownea clade in which this occurs, the adaxial petal involves, to a greater or lesser extent, the innermost whorls (stamens and carpel) as a whole (Tucker 2000b, 2002b). However, in *H. mimosoides*, *M. suaveolens*, *P. paraensis* and *P. speciosa*, we see that the adaxial petal only involves the carpel during the late stages of development (Figs. 8d, 10c, d, 12g, 14d), remaining so until pre-anthesis. This is a characteristic not previously observed in other ontogenetic studies for Detarioideae and is possibly a synapomorphy of the clade that includes *Heterostemon*, *Macrolobium* and *Paloue*. This does not occur in *Brownea* and *Browneopsis*, which have 1-2 stamens between the carpel and the adaxial petal. In *Ecuadendron*, with five stamens and four staminodes, it was observed that the adaxial petal surrounds the carpel and the stamens equally in the bud (present study). However, observations regarding this character are still necessary in the genus *Brachycylix* and it is also necessary to carry out a more comprehensive survey to verify whether this characteristic does not actually occur in other closely related taxa of Detarioideae.

Within the Brownea clade, there is vast variation in floral symmetry, and this is due to differences in initiation and heterochrony, especially in the whorls of the corolla and androecium. Actinomorphy or actinomorphic-like flowers predominates in two genera (*Brownea* and *Browneopsis*) and zygomorphy in five (*Brachycylix, Ecuadendron, Heterostemon, Macrolobium, Paloue*).

*B. longipedicellata* and *B. leucantha*, although appearing to be actinomorphic, are slightly zygomorphic due, mainly, to late processes in the development of the corolla (Fig. 2.2, 2.8). In both cases, simultaneous or almost simultaneous initiation patterns predominate for almost all whorls except the calyx (Table 2) and the organs of the same whorl generally develop equally. At anthesis, the petals are equal in size but acquire a slightly uneven arrangement. Different flowers from the same inflorescence may have slightly different symmetries. This is possibly due to pressure from adjacent organs and flowers. In the case of *B. leucantha*, these differences are barely noticeable when analyzing the inflorescence as a whole. The correlation between actinomorphy and erraticity in organogenesis has been considered by Tucker (1999). The flowers of *B. longipedicellata* have synorganization in bracteoles and stamens (Endress 2006). There is partial fusion of their bracteoles which, because they are also exceptionally large compared to the size of the flower, surround the inner whorls and structure the pseudotubular architecture of the flower at anthesis. In certain *Brownea* species, such as *Brownea macrophylla* Linden ex Mast., this structure is less evident and the flowers are more open. In *P. speciosa*, the petals also remain almost erect at anthesis, although they are not structured by the pair of bracteoles.

In the Brownea clade, zygomorphy predominates and is most intense in *Brachycylix* and in the clade comprising *Macrolobium*+*Heterostemon*+*Paloue*. In relation to the taxa studied in this clade, we see that the zygomorphy of the corolla mainly involves processes of heterochrony during intermediate and late stages of floral development, notably in relation to the heteromorphy of the adaxial petal in *P. paraensis* (wider) and *P. speciosa* (narrower), and the reduction of the two abaxial petals in *H. mimosoides*. The lateral and abaxial petals in *M. suaveolens* generally do not form and, when they do, they are suppressed at an early stage of development. In the case of the androecium, the zygomorphy is present from organogenesis, with unidirectional initiation, later accompanied by heterochrony.

*M. suaveolens* exhibits patterns of corolla and androecium reduction that are, in some respects, similar to those of *P. paraensis*, *P. speciosa* and *H. mimosoides*. However, the disruptive impact of bracteoles on the development of subsequent whorls is what differentiates *M. suaveolens* from the others. The vast majority of taxa whose floral meristems were identified as being of the omega type have strongly zygomorphic flowers, with the exception of *Isoberlinia* and *Tessmannia* (Tucker 2002a).

*Hypanthium and pollination strategies*—Most Detarioideae have hypanthia, which are often internally lined with nectariferous tissue (Bruneau et al. 2014; Cowan and Polhill 1981a). In general, the hypanthium is tubular, although its form varies considerably among the taxa examined (Fig. 2.5, 2.7, 2.18, 2.21, 2.28, 2.31).

In the Brownea clade, ornithophily—primarily involving hummingbirds—is recorded in many species of *Brownea* (Arroyo 1981; Klitgaard 1991) and *Paloue* (Arroyo 1981). Chiropterophily has been documented in *Browneopsis* (Arroyo 1981; Fleming et al. 2009; Klitgaard 1991; Knudsen and Klitgaard 1998), *Paloue* (Arroyo 1981; Banks and Rudall 2016; Carvalho 1961; Fleming et al. 2009; Rodrigues et al. personal observation), and is inferred for *Ecuadendron* (Neill 1998). Phalaenophily has been recorded in *Browneopsis* (Arroyo 1981; Klitgaard 1991; Knudsen and Klitgaard 1998) and psychophily in *Macrolobium* (van Dulmen 2001). However, there is currently no data on the pollination strategies of *Brachycylix* and *Heterostemon*.

*B. longipedicellata* and *P. speciosa* have a more typical tubular hypanthium structure, with nectariferous tissue concentrated in the basal region of the tube (Fig. 15a–c, n, o). Both species have a gynoecium inclined toward the adaxial side of the flower, which emerges through the opening of the staminal ring in *B. longipedicellata*, a strategy that possibly aims to facilitate pollinator access to hypanthium. Furthermore, flowers of both species are pseudotubular and red. The formation of at least partially pseudotubular flowers has sometimes occurred independently in different subfamilies of Fabaceae, such as in Cercidoideae (*Lysiphyllum cunninghamii* (Benth.) de Wit), Detarioideae (genera *Barnebydendron, Schotia* and some species of *Eperua* and *Humboldtia*) (Kiepiel et al. 2022; ter Steege et al. 2023; Warwick et al. 2008), Dialioideae (*Zenia*) (Falcão et al. 2023), Caesalpinioideae (*Colvillea*, some species of *Delonix*, *Jacqueshuberia*, among others) (Banks 1997; Lewis 2005) and Papilionoideae (*Erythrina, Camptosema, Butea, Swainsona* Salisb., *Dahlstedtia*, among others) (Ferreira 2010; Jusaitis 1994; Missagia et al. 2014; Souza et al. 2024; Tandon et al. 2003), and is commonly associated with ornithophily, also evidenced by another parallelism in most of these lineages, such as the red colour of the flowers which are odorless, otherwise uncommon in Fabaceae (Arroyo 1981; Franklin 1999; Lewis et al. 2005).

The hypanthium of *B. leucantha* is deep, but relatively narrow, forming a crescent shape around the stipe. *P. paraensis* has the shallowest hypanthium among the six species, making it exposed to floral visitors, and its nectariferous tissue is relatively extensive compared to the others (Fig. 15g–j), producing abundant amounts of nectar, which is consistent with large pollinators with high energy needs such as bats (Arroyo 1981; Carvalho 1961; Rodrigues et al. personal observation). In *P. paraensis*, the gynoecium leans slightly to the adaxial side, as in *P. speciosa*, but the flower of *B. leucantha* greatly restricts access to the hypanthium, narrowing the opening of the staminal ring around the carpel, so that only visitors such as bats and moths would be able to access it without damaging the flower (Knudsen and Klitgaard 1998). Both species present white and fragrant flowers.

*H. mimosoides* has large, purple, and odorless flowers with nectar guides on the adaxial petal and receptacle, as well as a long and narrow hypanthium, while *M. suaveolens* has small and white/green and fragrant flowers with a short and gibbose hypanthium. However, both have similar strategies of restricting access to hypanthium, in which the ovary leans to the abaxial side, next to the stamens, a common characteristic in Detarioideae (Cowan and Polhill 1981a). Both have different morphological characteristics that could be related to adaptation for pollination by Lepidoptera of different sizes. The nectariferous tissue of *H. mimosoides* is concentrated at the base of its hypanthium, making access to the nectar very difficult for other types of visitors (Fig. 15d–f) and favoring large Lepidoptera with long proboscises, although access by hummingbirds with long tongues may also be possible. The claw of the adaxial petal of *M. suaveolens* is strongly auriculate, forming a narrow duct that leads to the hypanthium, which is probably the only way to access it, due to the carpel closing the hypanthium opening. There is a record of butterfly pollination for one species of *Macrolobium* (van Dulmen 2001), but the white petals of the genus may indicate a greater association with nocturnal pollinators such as moths (Weberling 1989).

It is important to emphasize that the anatomical studies carried out on the hypantial structure of *B. longipedicellata*, *H. mimosoides*, *P. paraensis* and *P. speciosa* were made with flower buds close to anthesis, and there may be modifications in the nectariferous tissue of the flower in anthesis regarding what is illustrated in Fig. 15.

*Final considerations—*This study provides new data on floral development within the Brownea clade, a morphologically diverse group within Detarioideae that has previously received limited attention in terms of ontogenetic research. The findings contribute to discussions on the factors influencing inflorescence morphology and meristic and symmetry variation on flowers, from an evolutionary standpoint.

A key factor in the morphological differentiation of inflorescences is variation in plastochrony between meristems. Notably, *B. longipedicellata* displays basipetal sequential development, a distinctive morphological trait. Bracteoles play a critical role in the evolutionary history of Detarioideae, particularly within the tribe Amherstieae. They not only contribute to floral architecture but also influence floral development in several lineages. The pressure exerted by the bracteoles, combined with the unidirectional initiation of the androecium and heterochrony of floral organs, are primary factors behind meristic reduction. However, the cause of the ontogenetic loss of the stamen opposite the adaxial petal in various lineages remains unclear. This reduction has led to the clade with *Heterostemon* + *Paloue* + *Macrolobium* presenting the adaxial petal laterally enveloping the carpel through the space between the stamens and the carpel—a unique characteristic within Detarioideae, which may represent a synapomorphy for the clade.

Finally, the remarkable diversity of pollination strategies among the studied taxa is noteworthy. Even species adapted to the same type of pollinator exhibit distinct differences in their pollination mechanisms. The anatomical structure of the nectariferous tissue is similar across species, but the variation in the shape, depth, and width of the hypanthium, as well as the methods by which the pollinator accesses it, highlight the extensive array of adaptations present within the clade.

## Supplementary Information

Below is the link to the electronic supplementary material.

Supplementary Material 1
