# Supplementary material for: Floral and inflorescence ontogeny in the Brownea clade (Fabaceae: Detarioideae):insights into a morphologically diverse lineage of legumes
Source: J Plant Res. 2026 Jul 20;139(5):805–40. doi: 10.1007/s10265-026-01723-2 (PMC13588895; doi:10.1007/s10265-026-01723-2)
Supplement: Supplementary file 1 [file 10265_2026_1723_MOESM1_ESM.docx]

**Floral and inflorescence ontogeny in the Brownea clade (Fabaceae: Detarioideae): Insights into a morphologically diverse lineage of Legumes**

Ana Tereza Novaes Parga Rodrigues^1, 3*^, Marcus José de Azevedo Falcão^1, 2, 4^, and Vidal de Freitas Mansano^1, 5^

¹Instituto de Pesquisas Jardim Botânico do Rio de Janeiro, DIPEQ. Rua Pacheco Leão 915, Jardim Botânico, Rio de Janeiro, RJ, 22460–030, Brazil; ²Universidade do Estado do Rio de Janeiro, IBRAG-DBV. Rua São Francisco Xavier, 524, Maracanã, Rio de Janeiro, RJ, 20550-900, Brazil.

³ana.tnovaes@gmail.com; <https://orcid.org/0000-0002-1235-1655>

^4^jrfalcao2010@hotmail.com;<https://orcid.org/0000-0001-8337-5112>

^5^vidalmansano@gmail.com;<https://orcid.org/0000-0002-7204-0744>

*Corresponding author: [ana.tnovaes@gmail.com](mailto:ana.tnovaes@gmail.com)

Table S1: Detarioideae taxa with omega-type floral meristem and floral reduction events.

| **Taxa** | **S** | **P** | **Initiated P** | **Stamen** | **Initiated st** | **Staminode** | **Tribe** |
| --- | --- | --- | --- | --- | --- | --- | --- |
| *Sindora klaineana* Pierre ex Pellegr.*⁴* | 5 | 1(4) | 5 | 2 | 10 | 8 | Detarieae |
| *Tessmannia africana* Harms*²* | 5 | 5 | 5 | 10 | 10 | 0 | Detarieae |
| *Afzelia quanzensis* Welw.*³* | 5 | 1(4) | 5 | 7 | 9 | 0 | Afzelieae |
| *Macrolobium suaveolens* Spruce ex Benth. | 5 | 1 | 3–5 | 3 | 5–7 | 0 | Amherstieae |
| *Paramacrolobium*  *coeruleum* (Taub) J. Léonard*³* | 5 | 1(4) | 5 | 3 | 9 | 6 | Amherstieae |
| *Neochevalierodendron*  *stephanii* (A. Chev.) J. Léonard*³* | 5 | 3 | 5 | 10 | 10 | 0 | Amherstieae |
| *Hymenostegia klainei* Pierre ex Pellegr.*²* | 5 | 3(2) | 5 | 10 | 10 | 0 | Amherstieae |
| *Isoberlinia angolensis* (Welw. ex Benth.) Hoyle & Brenan*²* | 5 | 5 | 5 | 10 | 10 | 0 | Amherstieae |
| *Berlinia grandiflora* (Vahl) Hutch. & Dalziel*³* | 5 | 1(4) | 5 | 10 | 10 | 0 | Amherstieae |
| *Anthonotha crassifolia* (Baill.) J. Léonard*³* | 5 | 1(4) | 5 | 3 | 10 | 0 | Amherstieae |
| *Microberlinia bisulcata* A. Chev.*²*  *Microberlinia brazzavilensis* A. Chev.*²* | 4-5 | 5 | 5 | 10 | 10 | 0 | Amherstieae |
| *Brachystegia boehmii* Taub*¹*  *Brachystegia tamarindoides* Welw. ex Benth.*¹* | 5 | 0 | 4 | 10 | 10 | 0 | Amherstieae |
| *Julbernardia pellegriniana* Troupin*⁴* | 5 | 3(2) | 5 | 10 | 10 | 0 | Amherstieae |
| *Aphanocalyx djumaensis* (D. Wild.) J. Léonard*¹* | 1–3 | 1(0–2) | 3 | 10 | 10 | 0 | Amherstieae |
| *Bikinia durandii* (F. Hallé & Normand) Wieringa*¹* | 1–5 | 1 | 3 | 10 | 10 | 0 | Amherstieae |
| *Tetraberlinia tubmaniana* J. Léonard*³* | 5 | 3(2) | 5 | 10 | 10 | 0 | Amherstieae |
| *Gilbertiodendron klainei* (Pierre ex Pellegr.) J. Léonard*³* | 5 | 1(4) | 5 | 3 | 10 | 0 | Amherstieae |

Notes: Sources: (¹) = Tucker 2000a, (²) = Tucker 2002a, (³) = Tucker 2002b, (⁴) = Tucker 2003c; In column P, the numbers of reduced petals are indicated in parentheses; Species analysed in the present study have a highlighted row; (S) = sepal, (P) = petal, (st) = stamens.

Table S2: Detarioideae taxa with circular-type floral meristem and floral reduction (or rare proliferation) events.

| **Taxa** | **S** | **P** | **Initiated P** | **Stamens** | **Initiated st.** | **Staminode** | **Tribe** |
| --- | --- | --- | --- | --- | --- | --- | --- |
| *Schotia afra* Thunb.*³*  *Schotia brachypetala* Sond.*³*  *Schotia latifolia* Jacq.*³* | 5 | 5 | 5 | 10 | 10 | 0 | Schotieae |
| *Barnebydendron*  *riedelii* (Tul.) J. H. Kirkbr*¹* | 5 | 3 | 5 | 10 | 10 | 0 | Barnebydendreae |
| *Goniorrhachis marginata* Taub.*⁵* | 4 | 5 | 5 | 10 | 10 | 0 | Barnebydendreae |
| *Colophospermum mopane* (J. Kirk ex Benth.) J. Kirk ex J. Léonard*⁸* | 2 | 0 | 0 | 20 | 20 | 0 | Detarieae |
| *Copaifera langsdorfii* Desf.*⁴* | 5 | 0 | 5 | 10 | 10 | 0 | Detarieae |
| *Hymenaea verrucosa* Gaertn.*⁶* | 5 | 3(2) | 5 | 10 | 10 | 0 | Detarieae |
| *Brodriguesia santosii* R. S. Cowan*⁷* | 5 | 5 | 5 | 10 | 10 | 0 | Afzelieae |
| *Amherstia nobilis* Wall.*¹* | 5 | 3(2) | 5 | 9 | 9 | 0 | Amherstieae |
| *Tamarindus indica* L.*¹* | 5 | 3(2) | 5 | 3 | 9 | 0 | Amherstieae |
| *Brownea latifolia* Jacq.*¹* | 5 | 5 | 5 | 10–11 | 10–11 | 0 | Amherstieae |
| *Brownea longipedicellata* Huber | 5 | 5 | 5 | 10–11 | 10–11 | 0 | Amherstieae |
| *Brownea leucantha* Jacq. | 5 | 5 | 5 | 10–11 | 10–11 | 0 | Amherstieae |
| *Heterostemon mimosoides* Desf. | 5 | 3(2) | 5 | 3 | 9 | 6 | Amherstieae |
| *Paloue paraensis* (Ducke) Redden  *Paloue speciosa* (Ducke) Redden | 5 | 5 | 5 | 3 | 9 | 6 | Amherstieae |
| *Crudia acuminata* Benth.*²* | 5 | 0 | 5 | 10 | 10 | 0 | Amherstieae |
| *Cynometra webberi* Baker F*.³* | 5 | 5 | 5 | 10 | 10 | 0 | Amherstieae |

Notes: Sources: (¹): Tucker 2000b, (²) = Tucker 2001a, (³) = Tucker 2001b, (⁴) = Pedersoli et al. 2010, (⁵) = Prenner and Cardoso 2017, (⁶) = Kochanovski et al. 2018, (⁷) = Citeli et al. in prep, (⁸) = Krüger et al. 1999; In column P, the numbers of reduced petals are indicated in parentheses; Species analysed in the present study have a highlighted row; (S) = sepal, (P) = petal, (st) = stamens.
